# Estrogen and obesity synergistically suppress protein S via HIF1**α**, enhancing thrombosis potential

**DOI:** 10.1172/JCI193976

**Published:** 2025-11-17

**Authors:** Mohammad A. Mohammad, Narender Kumar, Sonali Ghosh, Ashley Paysse, Claudia Leonardi, Vijaya Pilli, Ma Lorena Duhaylungsod, Eric Lazartigues, Diana C. Polania-Villanueva, Sadaf Nouman, Logan A. Barrios, Rima Chattopadhyay, Rafika Yasmin, Alaina Guilbeau, Manoj Kumar, Tina Nguyen, Jovanny Zabaleta, Li Li, Luis Del Valle, Mallory T. Barbier, Samarpan Majumder, Laurent O. Mosnier, Rinku Majumder

**Affiliations:** 1 Department of Interdisciplinary Oncology,; 2 Department of Orthopedics,; 3 Cardiovascular Center of Excellence,; 4 Department of Genetics, and; 5 Department of Pathology, Louisiana State University Health Sciences Center (LSUHSC), New Orleans, Louisiana, USA.; 6 Department of Translational Medicine, Scripps Research, La Jolla, California, USA.

**Keywords:** Hematology, Vascular biology, Coagulation, Hypoxia, Thrombosis

## Abstract

Venous thromboembolism (VTE) is a leading cause of morbidity and mortality, with risk heightened in premenopausal women with obesity or use estrogen-based oral contraceptives. When both risk factors are present, the thrombosis risk increases substantially. Protein S (PS), an essential anticoagulant cofactor, is downregulated by both estrogen and obesity, but the molecular basis for this suppression remains poorly defined. We investigated the effect of estrogen and obesity on PS expression using plasma samples from 157 women stratified by BMI and contraceptive use, alongside 40 mice categorized as lean or obese with or without estrogen pellet treatment. The levels of PS were reduced by either estrogen or obesity alone, and the combined effect increased thrombin generation. In HepG2 hepatocytes, hypoxic conditions (1%–10% O_2_) mimicking obesity, with or without 17 β-estradiol, suppressed *PROS1* transcription and promoter activity. ChIP confirmed direct binding of hypoxia-inducible factor 1α (HIF1α) to the *PROS1* promoter, repressing gene expression. These findings define a mechanistic pathway through which estrogen and obesity converge to suppress PS synthesis, providing insight into the elevated thrombosis risk observed in women with obesity using estrogen-based contraceptives.

## Abstract 2

This study reveals how estrogen and obesity together reduce Protein S levels, increasing blood clot risk in premenopausal women using hormonal contraceptives.

## Introduction

Thrombosis is a leading cause of morbidity and mortality globally, with venous thromboembolism (VTE) responsible for 60,000–100,000 deaths annually in the United States. Two prevalent risk factors, obesity and estrogen-based oral contraceptives (OCAs), contribute substantially to VTE in premenopausal women (1–3) The prothrombotic effect of estrogen-based OCAs is well established (4, 5), in part due to reductions in the anticoagulant protein S (PS) (6, 7). PS, primarily expressed in hepatocytes, plays a crucial role in anticoagulation, and its deficiency is associated with acquired hypercoagulability (8–10). Estrogen suppresses PS gene transcription via estrogen receptor α (ERα) (6, 11), leading to lower plasma PS levels and a 3-fold increased risk of VTE in women who take OCAs (12, 13). Notably, while studies indicate that PS levels are lower in premenopausal compared with levels in postmenopausal women, the association between moderately reduced PS levels and VTE risk remains a matter of debate (14–16). Large population studies, such as Multiple Environmental and Genetic Assessment (MEGA) (17), suggest that only severe PS deficiency (<33% of normal levels) is definitively linked to VTE, leaving open the question of whether more modest reductions, as seen in individuals taking estrogen, contribute to hypercoagulability. However, a key limitation of the MEGA study was its inability to distinguish between premenopausal and postmenopausal women using OCAs.

Obesity, defined as a BMI of 30 kg/m^2^ or higher, is a well-recognized independent risk factor for VTE (18), increasing the risk by up to 6-fold (3, 19–22). Obesity has been linked to decreased plasma PS levels, which may contribute to hypercoagulability, although its direct role in VTE remains to be fully elucidated (20, 23, 24). Alarmingly, the combination of obesity and estrogen-based OCA use increases VTE risk up to 24-fold, representing serious public health concerns (2, 23, 25–27). Obesity is a chronic hypoxic state (28–32), with hepatic hypoxia being a key, yet often-overlooked, feature contributing to its prothrombotic effects (29).

We previously demonstrated that hepatic hypoxia stabilizes hypoxia-inducible factor 1α (HIF1α), leading to reduced PS expression and an increased risk of VTE (33). Emerging evidence indicates that hepatic oxygen levels in individuals with obesity are greatly reduced, and studies have reported that the levels of hypoxia in both obese humans (34–36) and obese mice (37) reached an oxygen concentration of approximately 2%. However, we also acknowledge that hypoxia is an experimental surrogate and not necessarily a direct representation of the condition in human obesity. Here, in a cohort of obese and nonobese women with and without OCA use, lean and obese mice with estrogen supplementation, and HepG2 liver cells, we investigate how estrogen and obesity synergistically downregulate PS expression. Our findings suggest that obesity-associated hepatic hypoxia and estrogen act in concert to suppress PS expression, offering mechanistic insight into the heightened VTE risk in women with obesity taking estrogen-based OCAs.

## Results

### Estrogen and obesity increase thrombin generation in human plasma.

**Figure 1:**
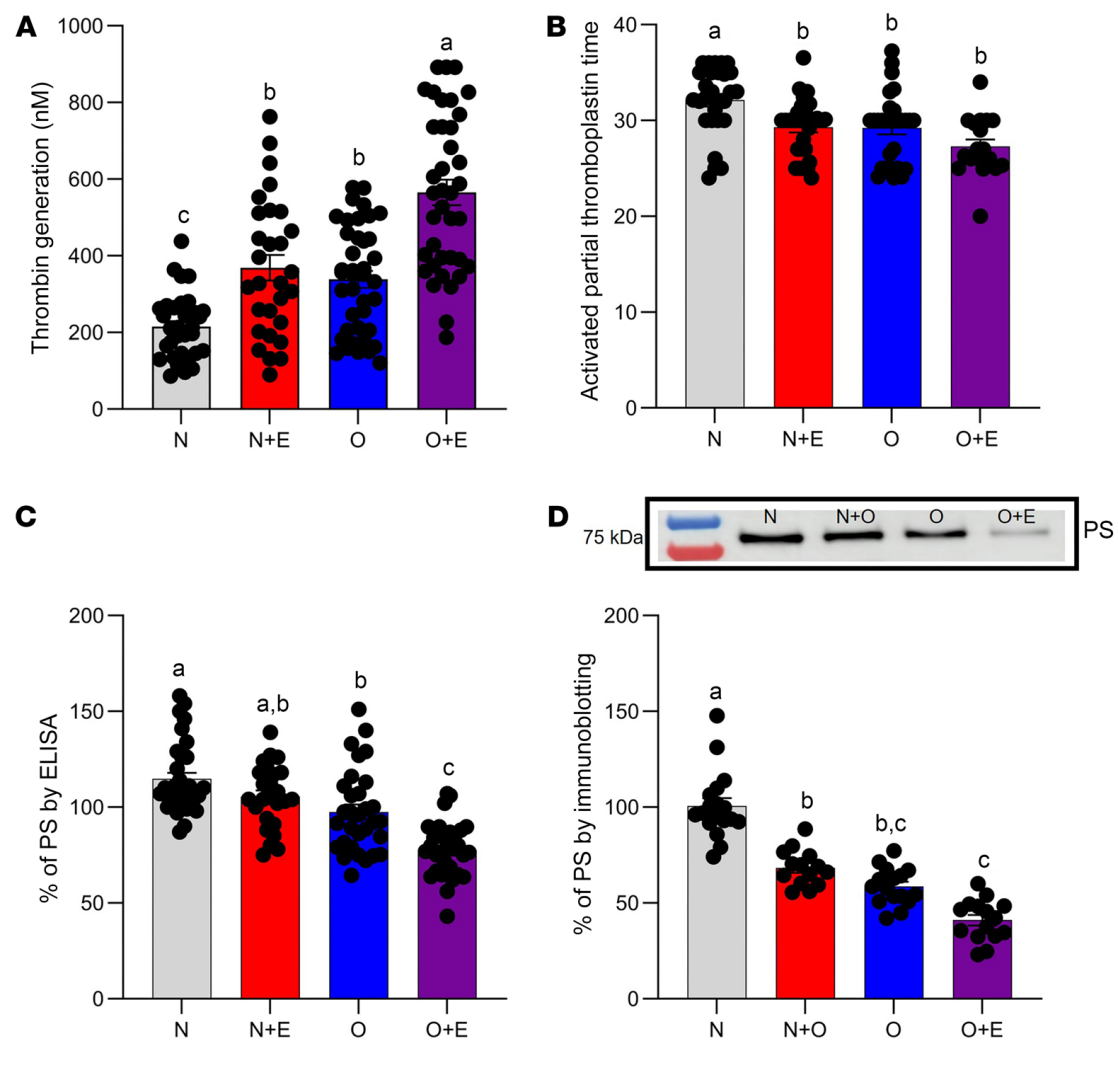
Estrogen and obesity downregulate PS in human plasma. The effects of estrogen and obesity on plasma from female participants (*n* = 157) grouped as N, N+E, O, or O+E were determined by TGA (**A**), aPTT (**B**), ELISA (**C**), and immunoblotting (**D**). ^a,b,c,d^Least squares means with adjusted for age as a covariate, with unique superscripts indicate significant differences between all female participants (*P* < 0.05). Pairwise comparisons of least squares means were adjusted using the Bonferroni method to correct for multiple comparisons. All data are presented as the mean ± SEM and are representative of 3 independent experiments. The color red represents N+E participant, blue represents O participants, and purple represents O+E participants.

PS prevents thrombin formation by acting as a cofactor for activated protein C and tissue factor pathway inhibitor and by directly inhibiting factor IXa, which subsequently inhibits thrombin generation (38–40). Therefore, we hypothesized that thrombin generation is affected in plasma of individuals who take estrogen-based OCAs and/or obese (hypoxic) individuals. Consequently, we collected plasma samples from premenopausal women with diverse demographic characteristics (Table 1) and grouped these women into 4 categories: normal (N), normal plus estrogen (N+E), obese (O), and obese plus estrogen (O+E). Measurement of peak thrombin by thrombin generation assay (TGA) (Figure 1A) showed an increase in thrombin formation by 1.5-fold in O women and 1.7-fold in N+E women, respectively. The combined effects of estrogen and obesity (Figure 1A) showed a substantial 2.7-fold increase in thrombin generation in O+E women. Additionally, supplementation of anti-PS antibody in normal plasma increased peak thrombin levels by approximately 2-fold (Supplemental Figure 1A; supplemental material available online with this article; https://doi.org/10.1172/JCI193976DS1), thereby reinforcing the effect of PS in thrombin generation and clotting time. Although activated partial thromboplastin time (aPTT) is primarily used in clinical settings to monitor anticoagulation therapy, we used it alongside TGA to achieve a more comprehensive assessment of coagulation status in plasma samples. Our results showed that the clotting time (Figure 1B) remained largely unchanged in O women and N+E women. However, in O+E women, the clotting time was shortened by 1.6-fold (Figure 1B) when compared with the normal reference range of clotting times (36.3 ± 2.8 seconds to 40.8 ± 1.5 seconds), which suggest a hypercoagulable state in those women (Supplemental Figure 1B). Next, to investigate whether PS supplementation mitigates the prothrombotic effects of low PS levels in O+E women, we conducted a plasma thrombin generation assay with and without exogenous PS (300 nM). PS supplementation in O+E plasma reduced peak thrombin by approximately 70%, restoring it to levels comparable to those in normal plasma (Supplemental Figure 1C).

### Estrogen and obesity attenuate PS in human plasma.

Measurement of free PS by ELISA (Figure 1C) showed a moderate reduction of 9% in PS levels in N+E women and 13% in O women, respectively. The combined effect of estrogen and obesity resulted in a 32.5% reduction in PS concentrations in plasma of O+E women (Figure 1C). We observed a similar trend in the reduction of PS expression (Figure 1D), reaching 32% in N+E women, 42% in O women, and a significant 60% in O+E women, respectively (Table 2). Moreover, we confirmed the inverse relationship between PS downregulation and thrombin generation (Supplemental Figure 1D) by measuring the correlation coefficient between the percentage of free PS and peak thrombin in O+E women, with a substantial negative Pearson correlation (*r*) value of –0.3782.

### Estrogen and obesity increase thrombin generation in mouse plasma.

**Figure 2:**
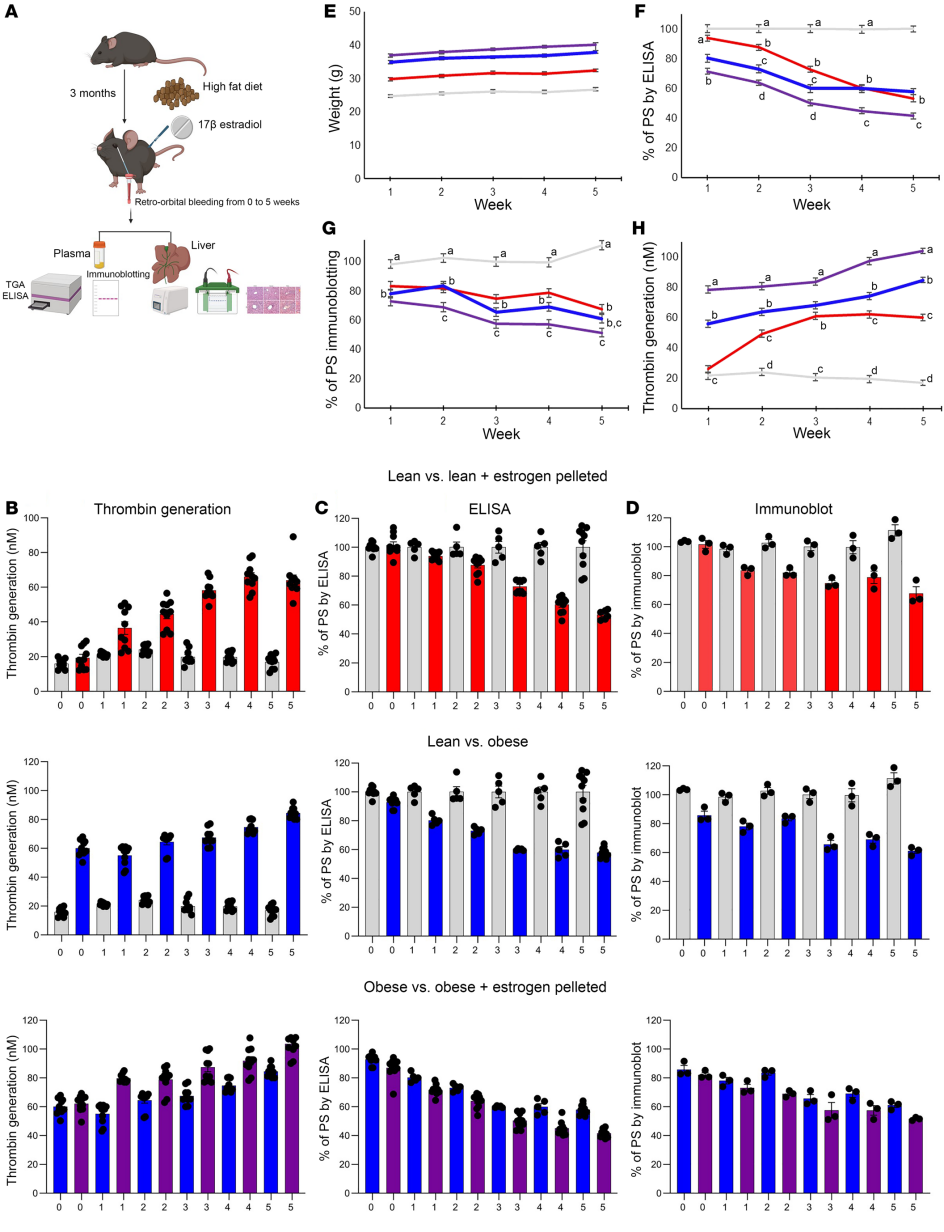
The effect of estrogen and obesity in a C57BL/6 mouse model. (**A**) Schematic illustration of the experimental design. The effects of estrogen and obesity on isolated plasma from female mice (*n* = 40) grouped as lean (L), lean plus estrogen pelleted (L+P), obese (O), and obese plus estrogen pelleted (O+P) were determined by TGA (**B**), ELISA (**C**), and immunoblotting (**D**). Overall significance of the entire mouse population emerged (*P* < 0.0001), indicating (**E**) variations in weight among the 4 mouse groups. Group comparisons were conducted for each week, revealing a significant overall group effect within each week (*P* < 0.0001) reflected on PS levels measured by ELISA (**F**), immunoblotting (**G**), and thrombin generation (**H**) over 5 weeks. Statistical analysis in **F**–**H** was performed using Bonferroni’s multiple-comparison adjustment, and distinctions among the 4 groups within each week are illustrated by distinct superscript lowercase letters a–d (*P* < 0.05). All data are presented as the mean ± SEM and are representative of 3 independent experiments.

Mice were also grouped into 4 categories (Figure 2A) — lean (L), lean plus estrogen pelleted (L+E), O, and O+E pelleted — to mimic the same conditions as the participants, and mouse plasma samples were analyzed by TGA to examine the effects of estrogen and hypoxia-associated obesity on thrombin generation over a period of 5 weeks. Measurement of peak thrombin (Figure 2B, top panel) showed an increase in thrombin generation that reached 3-fold in L+E mice, 4-fold in O mice, and a substantial 6-fold in O+E mice by the fifth week (Figure 2, B and H, top, middle, and bottom panels, and Table 3), respectively.

### Estrogen and obesity synergistically downregulate PS in mouse plasma.

Since mice lack the β-chain of C4BP in their plasma, PS exists predominantly as a free active form, and hence there is no distinction between free and total PS in mice (41). Therefore, PS from isolated plasma samples was analyzed by both ELISA and immunoblotting over a period of 5 weeks to determine the effects of estrogen and hypoxia on PS levels. Measurement of free PS by ELISA (Figure 2, C and F) showed a gradual reduction in PS concentration, reaching 40% in O mice (Figure 2C, middle panel), 50% in L+E mice (Figure 2C, top panel), and a substantial 62% in O+E mice (Figure 2C, bottom panel) by the fifth week compared with the control (L) mice. The expression of PS (Figure 2D) was also reduced by 30% in L+E mice (Figure 2D, top panel), by 40% in O mice (Figure 2D, middle panel), and by 51% in O+E mice (Figure 2D, bottom panel) compared with the control mice. Next, the correlation between adipose tissue and estrogen production was determined by comparing estrogen levels in control mice plasma (week zero) with levels at week 5 (Figure 2E). Measurement of 17 β-estradiol in plasma from mice showed a substantial increase in estrogen levels, reaching approximately 6.5-fold in O+E mice and 3.7-fold in L+E mice compared with L or O mice, respectively (Supplemental Figure 2).

### Estrogen and obesity downregulate PS expression in mouse liver.

**Figure 3:**
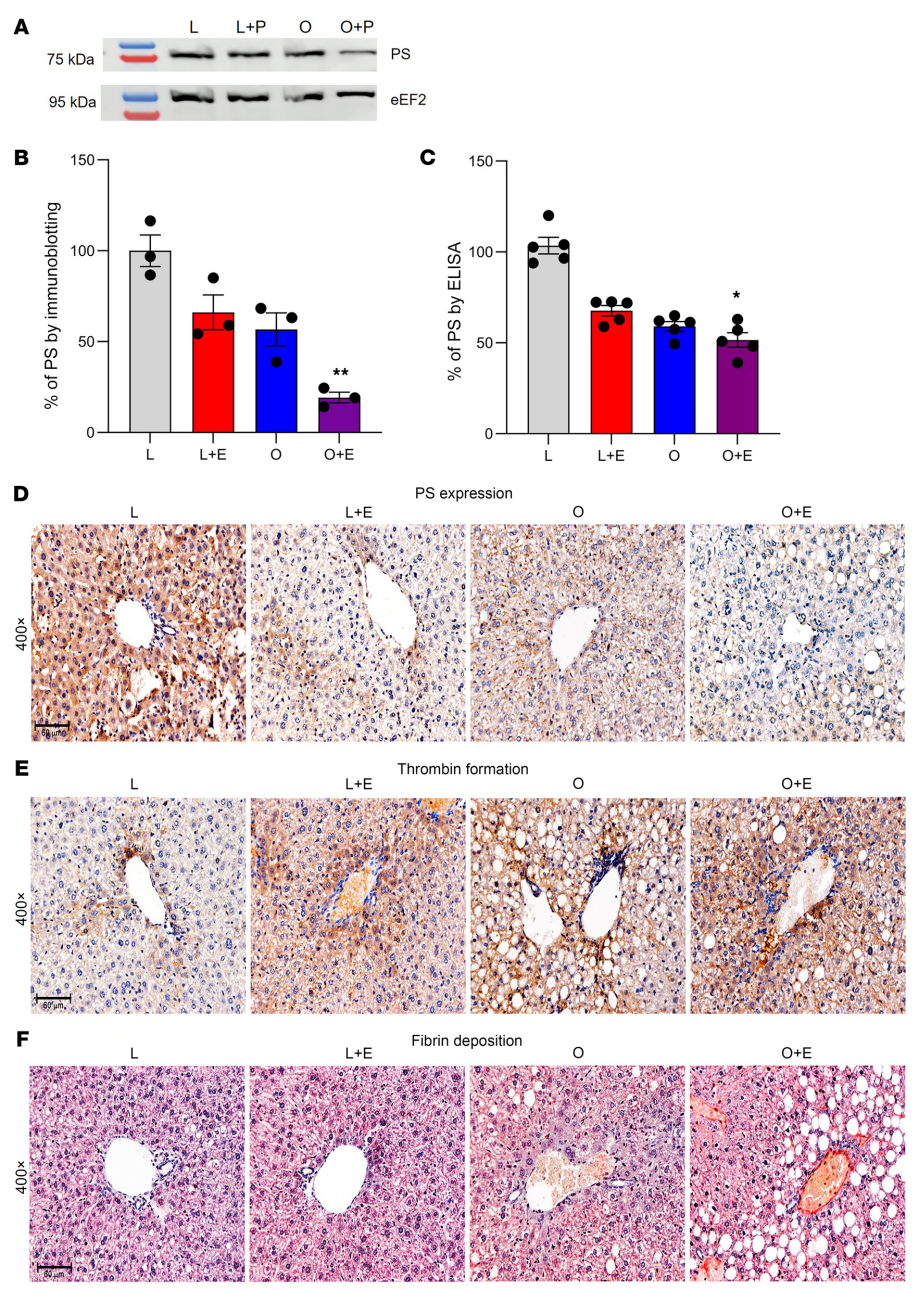
The combined effects of obesity and estrogen on the downregulation of PS in mouse liver. (**A**) Immunoblot showing the effects of estrogen and obesity on the expression of PS in different mice liver samples. eEF2, Eukaryotic elongation factor 2. Relative PS levels were determined by immunoblotting (**B**), ELISA (**C**), and IHC staining (**D**). Histological representation shows thrombin formation (**E**) and fibrin deposition (**F**) in mouse liver samples. Original magnification, ×400 (scale bars: 60 μm). *n* = 3 replicates of liver samples isolated from each mouse group. *P < 0.05 and **P < 0.01.

Since PS is synthesized primarily by the liver, and liver hypoxia is also associated with obesity, we used immunoblotting, ELISA, and IHC to examine the effects of estrogen and obesity on PS downregulation in liver tissues. Compared with the control (L), immunoblot quantification (Figure 3A) showed a 30%–40% reduction in PS expression in both L+E and O mice, whereas a significant 78% reduction in PS expression was observed in O+E mice (Figure 3B). Measurement of free PS by ELISA also showed a similar trend, reaching 30% in L+E mice, 35% in O mice, and 49% in O+E mice (Figure 3C). Furthermore, IHC analysis of PS expression (Figure 3D and Supplemental Figure 3A) also showed a substantial reduction in PS in both L+E mice (second panel) and O+E mice (fourth panel) compared with the L control mice (first panel). However, PS expression was slightly decreased in O mice (third panel) compared with the control and other 2 groups (Figure 3D). Furthermore, to determine the correlation between HIF1α expression with respect to the O and O+E groups, RNA was isolated from liver samples, and HIF1α was quantified by reverse transcription quantitative PCR (RT-qPCR). Compared with the control L mice, we observed a significant increase in *Hif1a* mRNA, reaching 50% in O+E mice and 25% in O mice, respectively (Supplemental Figure 4).

### Estrogen with obesity upregulates thrombin formation and fibrin deposition in mouse livers.

Next, we performed IHC to determine the effect of PS downregulation on thrombin formation. Analysis of mouse liver samples showed similar patterns in thrombin formation and intensity (Figure 3E and Supplemental Figure 3B), particularly at hepatocytes adjacent to the centrilobular vein in both L+E mice (Figure 3E, second panel) and O mice (Figure 3E, third panel) compared with control L mice. In O+E mice (Figure 3E, fourth panel), thrombin formation was substanially higher, particularly at the perivascular hepatocytes. Furthermore, analysis of fibrin with Martius Scarlet Blue staining (Figure 3F) showed no fibrin deposition in either the L or L+E mice. However, we observed newly deposited fibrin (yellowish color) in O and O+E mouse samples, in which fibrin occluded the lumen of numerous portals and centrilobular veins.

### RNA-Seq and differential gene expression.

**Figure 4:**
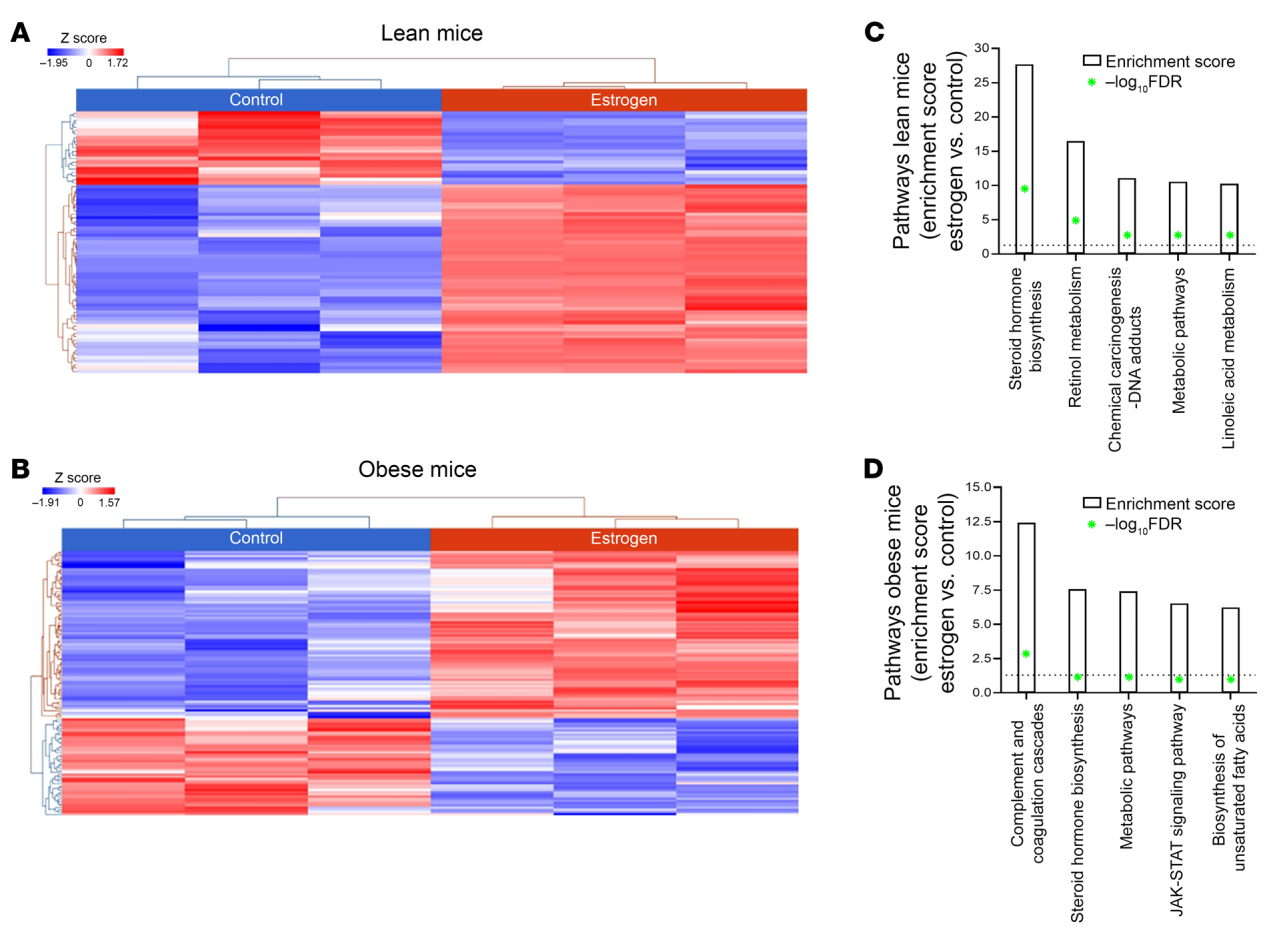
The effect of estrogen and obesity on the modulation of gene transcription. Heat map of differentially expressed genes identified in control (L) versus estrogen (L+E) mice (**A**) and O+E mice (**B**). Enrichment analysis of the top 5 pathways identified in estrogen versus lean (**C**) and obese (**D**) mice with a statistical significance of FDR ≤ 0.05. Heatmaps show differentially expressed genes and pathways that were identified by Partek Flow (DESEQ2). *n* = 3 replicates of liver samples isolated from each mouse group.

We performed whole-transcriptome RNA-Seq to identify genes regulated by estrogen and obesity in mouse liver. The heatmap generated showed substantial transcriptome differences between L+E (Figure 4A) and O+E (Figure 4B) mice compared with the L and O controls. The enrichment score of pathway analysis showed a significant upregulation in the steroid hormone biosynthesis pathway in estrogen-pelleted L+E mice (Figure 4C), whereas in O+E mice, we observed an upregulation in the complement and coagulation cascades (Figure 4D).

### Estrogen downregulates PS in HepG2 cells.

**Figure 5:**
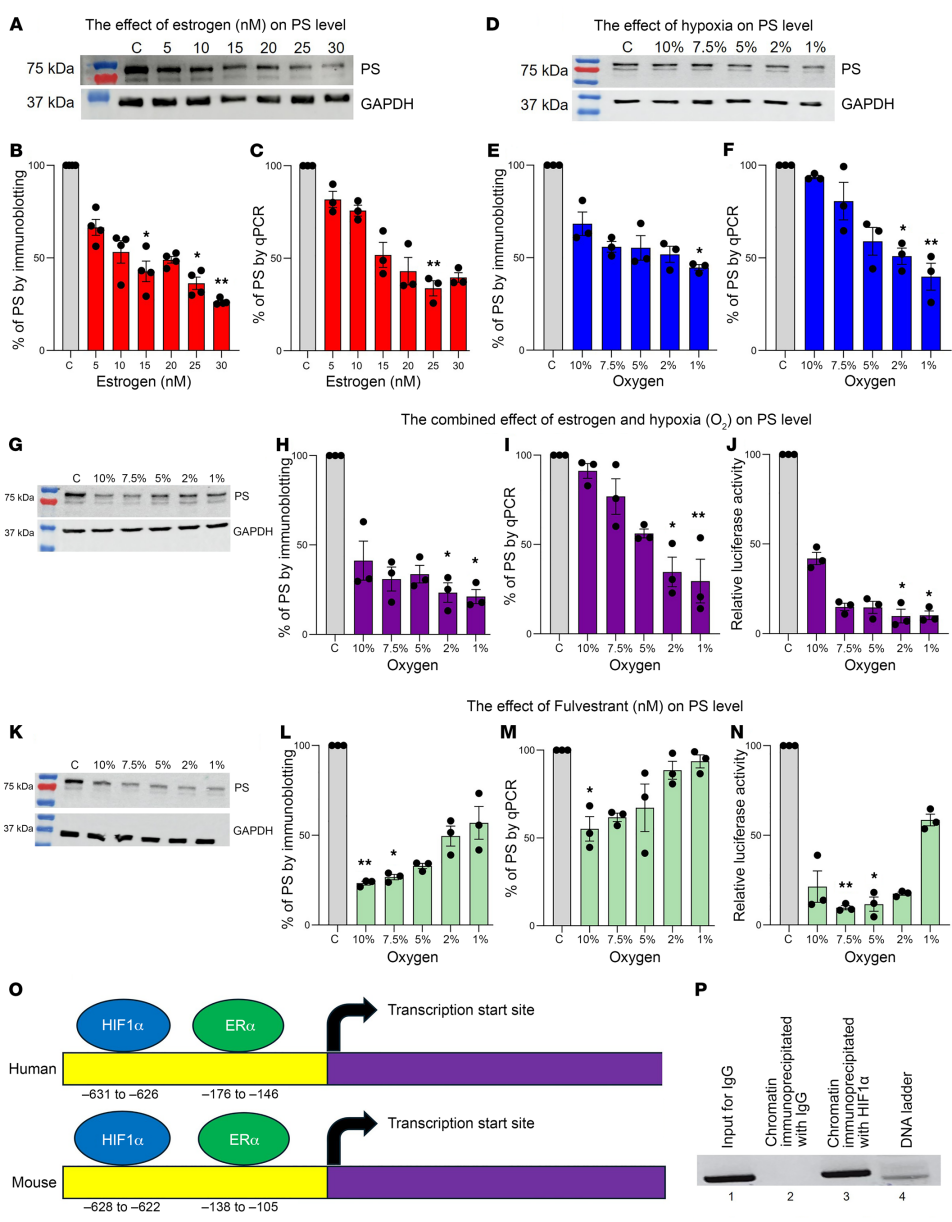
Estrogen and hypoxia downregulate PS expression and mRNA levels in HepG2 cells. (**A** and **D**) Estrogen-treated (5–30 nM) or hypoxia-induced (10%–1%) HepG2 cells were lysed and separated by SDS-PAGE, and the transferred proteins were probed with either PS or GAPDH to determine the effects on PS levels. Quantification of relative PS expression by immunoblotting (**B** and **E**) and mRNA levels by RT-qPCR (**C** and **F**). The combined effects of estrogen (25 nM) and oxygen concentrations (10%–1%) on the downregulation of PS were quantified (**G**), and relative PS expression was determined by immunoblotting (**H**), RT-qPCR (**I**), and luciferase assay (**J**). The reverse effect of 30 μM fulvestrant on PS levels was quantified (**K**), and relative PS expression was determined by immunoblotting (**L**), RT-qPCR (**M**), and luciferase assay (**N**). (**O**) Schematic diagram illustrating the possible binding sites of HIF1α on the PS promoter at position –631 to –626 in humans and at –628 to –622 in mice, respectively. (**P**) ChIP assay indicates the HIF1α binding site within the PS promoter. Lane 1: The presence of the PS amplicon indicates that HIF1α interacted with the PS promoter. Lane 2: Input DNA (isotype control). Lane 3: Absence of the PS promoter amplicon in the control antibody immunoprecipitation indicates the specificity of interaction of HIF1α with the PS promoter. Lane 4: DNA ladder. The bands were quantified by ImageJ software (NIH). The values shown are the mean ± SD of at least 3 independent experiments. Statistical significance was performed by 1-way ANOVA with Kruskal-Wallis correction, since data did not follow a normal distribution (*P < 0.05 and **P < 0.01). C, control.

To determine the effect of estrogen on PS expression, HepG2 cells were treated with various β-estradiol concentrations (5–150 nM) for 4 hours, and the expression of PS was measured by immunoblotting and RT-qPCR. Incubation of cells with lower estrogen concentrations (5–30 nM) (Figure 5A) resulted in a significant 60%–70% reduction in PS expression (Figure 5B) and mRNA levels (Figure 5C), particularly at 25–30 nM. At higher estrogen concentrations (50–150 nM), a similar pattern of reduction (Supplemental Figure 5A) reaching 70% was also observed in PS expression (Supplemental Figure 5B) and mRNA levels (Supplemental Figure 5C). We noticed that the maximal inhibitory effect on PS expression was achieved between 25–30 nM, and this concentration is also typically found in low-dose estrogen-containing contraceptive pills used by premenopausal women (42). Therefore, we conducted further experiments using an estrogen concentration of 25 nM.

### Hypoxia downregulates PS in HepG2 cells.

We have previously shown that PS expression is downregulated under hypoxic conditions (33). To simulate the hypoxic effects associated with obesity, hypoxia in HepG2 cells was induced by various oxygen concentrations (10%–1%) for 4 hours in a hypoxia chamber. Immunoblot analysis of hypoxia-induced cells (Figure 5D) resulted in a gradual reduction in PS expression (Figure 5E) and mRNA levels (Figure 5F), reaching 55%–60% at 1% O_2_, respectively. It was reported that cobalt chloride (CoCl_2_) stabilizes HIF1α, thereby mimicking hypoxia (43, 44). To independently confirm the effects of hypoxia on the downregulation of PS expression, we treated HepG2 cells with various concentrations (25–150 μM) of CoCl_2_ for 4 hours (Supplemental Figure 5D). Data analysis of immunoblots and RT-qPCR showed that 50 μM CoCl_2_ resulted in a significant 60%–65% reduction in both PS expression (Supplemental Figure 5E) and mRNA levels (Supplemental Figure 5F). However, higher CoCl_2_ concentrations did not exert significant inhibitory effects on the expression of PS.

### Estrogen and hypoxia synergistically downregulate PS in HepG2 cells.

To examine the combined effects of estrogen and hypoxia on the downregulation of PS, we treated HepG2 cells with 25 nM estrogen and incubated them under different hypoxic concentrations (10%–1% O_2_) for 4 hours. Immunoblot quantification (Figure 5G) showed a reduction in PS expression across all O_2_ concentrations, particularly at 1% O_2_, which resulted in a 78% reduction in PS expression (Figure 5H) and a 73% reduction in PS mRNA levels (Figure 5I). Next, the PS gene promoter region was subcloned into a luciferase reporter vector to examine the regulation of PS expression by HIF1α and ERα. Measurement of PS promoter activity also confirmed that both estrogen and hypoxia downregulated PS by 90% at 1% O_2_ (Figure 5J). Furthermore, by incubating the cells with 25 nM estrogen and 50 μM CoCl_2_ (Supplemental Figure 5G), we further confirmed the combined effect of hypoxia and estrogen on the downregulation of PS expression (Supplemental Figure 5H) and mRNA levels (Supplemental Figure 5I).

### HIF1α binds the PS gene promoter and targets its downregulation in HepG2 cells.

It has been reported that ERα represses PS expression by binding to the PS promoter at –176 to –146 in humans and at –138 to –105 in mice (6). HIF1α is a transcription factor that responds to hypoxia by inducing protective genes, but it also functions as a repressor of a small set of genes, including PS, as we have previously reported (33). However, the mechanism of PS gene repression by HIF1α has not, to our knowledge, been resolved at the molecular level. To identify the molecular mechanism, HepG2 cells were incubated for 4 hours with 50 μM CoCl_2_ to stabilize HIF1α and induce a hypoxia-like state. The binding of HIF1α to the PS gene promoter was confirmed by a ChIP assay (Figure 5O). The putative HIF1α binding site (ACTCG) in the promoter region of PS at position –631 to –626 in humans and –628 to –622 in mice (Figure 5P) was identified using ConSite and Genomatrix software. Note that these presumptive binding sites are in close proximity (~400–500 bp) to the established ERα binding sites (6, 45).

### Fulvestrant abolishes estrogen-mediated PS downregulation in HepG2 cells.

Fulvestrant is a *β*-estradiol analog and a selective antagonist that blocks ERα from binding to DNA and accelerates ERα degradation (46, 47). To reverse the effect of estrogen on the downregulation of PS, HepG2 cells were incubated with 25 nM estrogen under various hypoxic conditions (10%–1%), followed by 30 nM fulvestrant (Figure 5K). Both estrogen and hypoxia markedly reduce Protein S (PS) levels; therefore, we used both conditions to assess whether they contribute equally to PS downregulation. Fulvestrant-treated HepG2 cells showed a 56% increase in PS expression (Figure 5L), a 93% increase in mRNA levels (Figure 5M), and a 54% increase in luciferase promoter activity (Figure 5N) at 1% O_2_ compared with increases of 29% (Figure 5H), 18% (Figure 5I), and 36% (Figure 5J) without fulvestrant treatment, respectively.

### CAY10585 abolishes hypoxia-mediated PS downregulation in HepG2 cells.

To reverse the effects of hypoxia-mediated PS downregulation, we treated HepG2 cells with 25 nM estrogen under various hypoxic conditions (10%–1%), followed by 30 μM of the HIF1α inhibitor CAY10585 (Supplemental Figure 5J). Analysis of immunoblot and RT-qPCR data showed a 48% increase in PS expression (Supplemental Figure 5K) and a 52% increase in mRNA levels (Supplemental Figure 5L) at 1% O_2_ compared with 37% (Figure 5E) and 23% (Figure 5F) without CAY10585 treatment, respectively.

### RNA-Seq and differential gene expression.

Heatmap analysis of induced hypoxia (Supplemental Figure 6A), estrogen treatment (Supplemental Figure 6C), and the synergism of both treatments (Supplemental Figure 6E) showed that differential expression of genes was affected by estrogen and/or hypoxia treatment. Analysis of enrichment scores for hypoxia-induced HepG2 cells showed an upregulation in the HIF1α signaling pathway (Supplemental Figure 6B), while the metabolic pathways were upregulated in estrogen-treated cells (Supplemental Figure 6D). Moreover, the combined effects of estrogen and hypoxia resulted in the upregulation of both cell-cycle and metabolic pathways, with an enrichment score of 17.5 (Supplemental Figure 6F).

## Discussion

In this study, we provide a mechanistic explanation of how estrogen-based oral contraceptives and obesity elevate the risk of VTE. We demonstrate that estrogen and obesity independently reduced plasma PS levels and, more important, that estrogen and obesity acted synergistically, thereby amplifying the thrombosis risk. Using clinical plasma samples (Table 1 and Figure 1), mouse models (Figures 2–4), and in vitro HepG2 cell assays (Figure 5), we identified HIF1α as a key mediator of this downregulation of PS.

Our findings align with reports linking estrogen and obesity to increased thrombosis risk. Estrogen suppresses PS gene transcription via ERα (4, 6, 47), and obesity-related hypoxia stabilizes hepatic HIF1α, which also reduces PS level (18, 23). Although we initially described obesity-related hypoxia as a central mechanism in PS reduction, we acknowledge that obesity is a multifactorial metabolic disease and that hypoxia alone cannot fully encapsulate the complexity of obesity. Nevertheless, existing literature supports the biological plausibility of this link between hypoxia and obesity (48, 49). Adipose tissue hypoxia is well documented in individuals with obesity (50), and HIF1α activation occurs in hepatocytes of patients with nonalcoholic fatty liver disease, which is present in approximately 75% of individuals with obesity (51, 52). Furthermore, hypoxia-induced signaling via HIFs is associated with increased hepatic steatosis and coagulation pathway activation (53, 54). We further recognize that additional obesity-associated mediators, such as elevated circulating factor VIII and plasminogen activator inhibitor-1 (PAI-1), also contribute to thrombosis risk (18, 23, 55, 56). Notably, HIF1α regulates the expression of both tissue factor and PAI-1 (53, 57, 58), reinforcing the notion of a broader action of HIF1α in the dysregulation of coagulation in individuals with obesity, ultimately leading to increased thrombosis risk.

In support of our findings, thrombin generation assays of human plasma samples revealed an increase in thrombin formation in women with obesity and in those who take estrogen, with a significant 2.7-fold increase in women who are both obese and use estrogen (Figure 1, A and B). Similarly, in our mouse model, thrombin generation progressively increased over 5 weeks, reaching 6-fold elevation in obese mice supplemented with estrogen (Figure 2, B, bottom panel, and Figure 2F). These results suggest a hypercoagulable state in which PS depletion was a significant contributor. Consistent with these observations, we confirmed by ELISA and immunoblot analysis a considerable reduction in total (Figure 1D and Figure 2D, bottom panel) and free (Figure 1C and Figure 2C, bottom panel) PS levels in the O+E mouse group compared with either condition alone (Table 3). Notably, exogenous PS supplementation of plasma samples from this dual-factor group significantly attenuated thrombin generation (Supplemental Figure 1C), highlighting the direct contribution of PS depletion to hypercoagulability.

The liver is the primary source of PS; therefore, we used HepG2 cells to investigate the effects of estrogen and hypoxia on PS levels. Our in vitro studies confirmed that estrogen and hypoxia suppressed PS expression (Figure 5B and Supplemental Figure 5B) and reduced *PROS1* mRNA levels (Figure 5C and Supplemental Figure 5C). ChIP assays further revealed that HIF1α directly bound to the *PROS1* promoter and inhibited PS transcription (Figure 5, O and P). Notably, pharmacological inhibition of ERα with fulvestrant (Figure 5, K and N) or HIF1α with CAY10585 (Supplemental Figure 5, K and I) restored PS expression levels, suggesting that targeting these pathways could mitigate hypercoagulability. Although these findings underscore hypoxia’s effects in obesity-associated thrombosis, additional metabolic derangements such as chronic inflammation, insulin resistance, and dyslipidemia may contribute to thrombosis, and these possibilities warrant further investigation.

Histology of mouse liver tissues further supported our hypothesis (Figure 3D), revealing noticeable thrombin accumulation (Figure 3E) and fibrin deposition in the hepatic vasculature of O+E mice (Figure 3F). This accumulation suggests that PS downregulation not only affected systemic coagulation but also contributed to localized thrombotic events with potential implications for liver function and metabolic health.

RNA-Seq highlighted distinct transcriptomic alterations, particularly in O+E mice. Estrogen treatment upregulated steroid hormone biosynthesis pathways (Figure 4, A and C), while obesity-induced hypoxia activated complement and coagulation cascades (Figure 4, B and D). In O+E mice, these pathways were markedly enriched, reinforcing a synergistic effect of estrogen and obesity on dysregulation of coagulation. These findings emphasize the importance of integrating hormonal and metabolic risk factors into thrombosis risk assessment. Furthermore, our RNA-Seq analysis revealed that multiple miRNAs, such as miR-22, influence both ERα and HIF1α activity (59–61). Although these studies primarily focused on cancer cell signaling, they provide additional leads worth exploring in the context of hormone- and metabolically driven hypercoagulability.

The mechanistic framework we present here, namely, estrogen-mediated suppression of PS via ERα and obesity-induced HIF1α stabilization, may extend beyond the studied population. For instance, premenopausal and postmenopausal women receiving hormone replacement therapy (HRT) are another clinical population for whom our findings may be relevant. Hormonal fluctuations and the use of estrogen-based HRT elevate the risk for thrombosis, particularly for individuals with a personal or family history of VTE or thrombophilia (62). Although iron deficiency anemia and heavy menstrual bleeding are also implicated in menopausal thrombosis risk, suppression of anticoagulant proteins such as PS may further exacerbate hypercoagulability in these women. Our mechanistic understanding may therefore contribute to emerging risk stratification models for HRT-related thrombosis.

We used a comprehensive set of assays, including ELISA, TGA, aPTT, immunoblotting, IHC, and proteomics, to evaluate the effect of estrogen and hypoxia on PS levels, thrombosis potential, clotting time, and hepatic PS expression. These analyses yielded robust mechanistic explanations that will be useful for future assessments of the direct effect of estrogen and hypoxia on thrombosis susceptibility in venous thrombosis mouse models.

In summary, we identified the molecular basis for the heightened thrombotic risk of premenopausal women with obesity who use estrogen-based oral contraceptives. By identifying HIF1α as a central mediator of PS suppression, we have uncovered a potential therapeutic target. Strategies to modulate HIF1α activity, restore PS expression, or concurrently target additional coagulation regulators may mitigate thrombosis risk across diverse clinical scenarios.

## Methods

### Sex as a biological variable.

Only female participants and female mice were studied, given the biological relevance of estrogen treatment in women.

### Cell line.

HepG2 cells were obtained from the American Type Culture Collection (ATCC) and cultured in EMEM medium in Minimum Essential Medium (Corning; 10-009-CV) supplemented with 10% FBS (Thermo Fisher Scientific; A5670701). HepG2 (5 × 10^5^; WB, 5 × 10^4^; RT-qPCR, 25 × 10^5^; luciferase, 1 × 10^6^; ChIP) cells were seeded into a 6-well plate and allowed to attach overnight. The following day, the cells were washed twice with PBS and incubated with serum-free media overnight. To assess the combined effects of estrogen and/or hypoxia, the cells were prepared as described in Supplemental Methods.

### Blood collection.

The study cohort (Table 1) included a racially and ethnically varied population (Asian, African American/Black, White, Hispanic/Latino, other, and unknown) of 157 women, aged 18–45 years. The participants were divided into 4 groups: N (*n* = 44), N+E (*n* = 32), O (*n* = 42), and O+E (*n* = 39). Women in the estrogen groups who had been on estrogen-containing contraceptive pills within a period of 1 month to 3 years were included in the study (Supplemental Table 1). The participants with a BMI of 30 or higher were included in the obesity group. Venous blood was collected from volunteers into 3.2% buffered sodium citrate tubes. Platelet-poor plasma was separated immediately by centrifugation at 1,500*g* for 15 minutes at room temperature and then stored at –80°C for further assays.

### Thrombin generation assay.

Thrombin generation was performed exactly as described previously (63, 64) (see details in Supplemental Methods).

### Modified activated partial thromboplastin time assay.

aPTT was performed as described previously (63, 64) (see details in Supplemental Methods).

### Protein expression via immunoblotting.

Isolated human plasma was purified using the HAS/immunoglobulin Depletion Mini Spin Column (Thermo Fisher Scientific; A36366) to remove abundance immunoglobulins, while mouse plasma was purified using Multi Affinity Removal Spin Cartridge Mouse-3 (Agilent Technologies) according to the manufacturer’s instructions. The expression of PS was assessed by immunoblotting as described in Supplemental Methods.

### ChIP assay.

Cells were prepared using the Zymo-Spin ChIP Kit (Zymo Research; D5209) according to the manufacturer’s instructions. The cross-linked and sheared chromatin was precipitated with 5 μg HIF1α and by an isotype control IgG antibody (65, 66). The precipitated DNA was amplified using the mouse (67) and human primers listed in Supplemental Table 3.

### Animal model.

All animal procedures were performed according to protocols approved by the IACUC of LSUHSC. Six-week-old female mice (C57BL/6) were purchased from Charles River Laboratories (strain no. 000664) and were blindly randomized to 1 of 4 groups, with 10 mice in each group as detailed in Supplemental Methods. Each week, the blood was collected retro-orbitally for further assays (68).

### Quantitation of human and mouse PS by ELISA.

Human PS was quantified using the REAADS Free Protein S Antigen Test Kit (Diapharma), and murine PS was measured using our in-house–developed sandwich ELISA as described in Supplemental Methods.

### RT-qPCR assessment of gene expression.

HepG2 cells (5 × 10^4^) were seeded into 6-well plates overnight, and the cells were treated with various concentrations of β-estradiol (25–150 nM), or subjected to hypoxic conditions (10%–1%) or both treatments for 4 hours. The expression of *PROS1*, *HIF1A*, or *GAPDH* was quantified as detailed in Supplemental Methods.

### Effect of HIF1α and ERα on PS promoter activity.

To examine the synergistic effects of hypoxia and estrogen on PS expression, an 800 bp segment of the PS gene promoter was subcloned into the luciferase reporter pGL4 (Promega; E675A), and the activity of the promoter was measured as described in Supplemental Methods.

### Histology and IHC.

Isolated mice liver tissues were fixed in 10% buffered formalin and embedded in paraffin. H&E staining was performed for routine histopathological examination, while IHC was performed using the Vectastain ABC Elite Kit, according to the manufacturer’s instructions, as detailed in Supplemental Methods.

### RNA library preparation, sequencing, and analysis.

RNA quantification was performed using the Qubit RNA HS Assay kit (Invitrogen, Thermo Fisher Scientific; Q32855), and RNA quality was assessed with the Agilent 2100 Bioanalyzer (Agilent Technologies). The libraries were generated using Illumina Stranded Total RNA Prep with Ribo-Zero Plus library preparation kit (IIIumina; 20040526) according to the manufacturer’s instructions, as detailed in Supplemental Methods.

### Statistics.

Human and mice data were analyzed using SAS/STAT software 9.4 (SAS Institute), and significance was determined using the Bonferroni method as detailed in each figure legend. In the case of cell studies, statistical significance was performed using GraphPad Prism 9 (GraphPad Software) by 1-way ANOVA with Kruskal-Wallis correction. All data are expressed as the mean ± SEM, and experiments were repeated at least 3 times.

### Study approval.

The collection and use of clinical material for research purposes was approved by the IACUC of LSUHSC (approval no. 6324) and the IRB of LSUHSC (approval no. 912). Written informed consent was obtained for all human primary material.

### Data availability.

All data are available in the Supporting Data Values file and can be requested from the corresponding author. The sequencing data have been deposited in the Gene Expression Omnibus (GEO) database (GEO GSE285380).

## Author contributions

MAM performed the cell-based studies, analyzed the data, wrote the initial draft of the manuscript, and helped in the revision of the manuscript. NK performed the mouse studies, collected the plasma, performed all the experiments with mice and human plasma, and analyzed the data. SG performed mouse studies, collected plasma, performed all the experiments with mice and human plasma, and analyzed the data. AP, LDV, SK, and DPV performed phlebotomy, collected human plasma, and performed the thrombin generation assay, and AP performed the aPTT assays as well as immunoblotting. CL performed statistical analysis of the human and mouse studies. VSP initiated the study. SK, MLD, RC, and LB performed the TGA experiments using human samples. RY collected the data for the revised manuscript. MK, TN, and AG initially maintained the mouse colonies and initiated the mouse studies. EL helped with animal studies. JZ and LL performed RNA-Seq and analysis. LDV and MB performed and analyzed the IHC experiments. SM critically analyzed the cell data and reviewed the manuscript. LOM helped with implantation of estrogen pellets in mice, gave critical advice, and thoroughly reviewed and edited the initial and revised versions of the manuscript. RM conceptualized, designed, and directed the study, interpreted the results, and wrote the final manuscript, while supervising the study’s execution and securing funding and financial support. MAM, NK, and SG are co–first authors contributed equally to this work. Authorship order between them was determined based on manuscript preparation.

## Funding support

This work is the result of NIH funding, in whole or in part, and is subject to the NIH Public Access Policy. Through acceptance of this federal funding, the NIH has been given a right to make the work publicly available in PubMed Central.

- NIH grant R01HL151613 (to RM).
- NIH grant R01HL142975 (to LOM).

## Supplementary Material

Supplemental data

Unedited blot and gel images

Supporting data values

## Figures and Tables

**Table 2:**
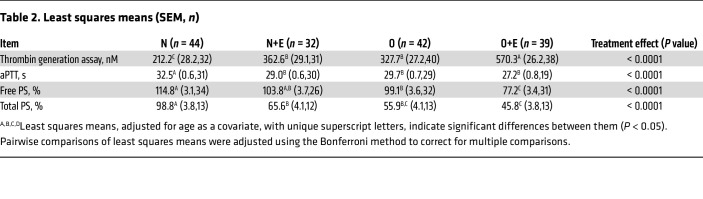
Least squares means (SEM, *n*)

**Table 3:**
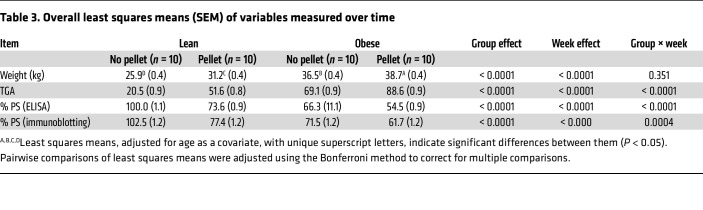
Overall least squares means (SEM) of variables measured over time

**Table 1:**
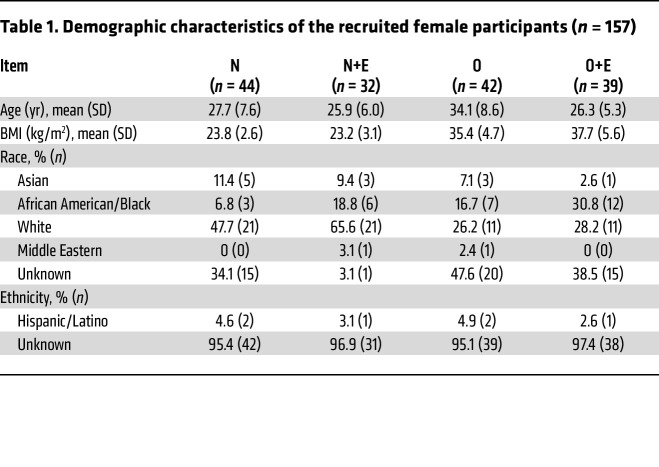
Demographic characteristics of the recruited female participants (*n* = 157)

## Version 1 11/17/2025

Electronic publication
