# Supplementary material for: Estrogen and obesity synergistically suppress protein S via HIF1α, enhancing thrombosis potential
Source: J Clin Invest. 2025 Nov 17;135(22):e193976. doi: 10.1172/JCI193976 (PMC12618064; doi:10.1172/JCI193976)
Supplement: Supplemental data [file jci-135-193976-s200.pdf]

Supplementary Figures

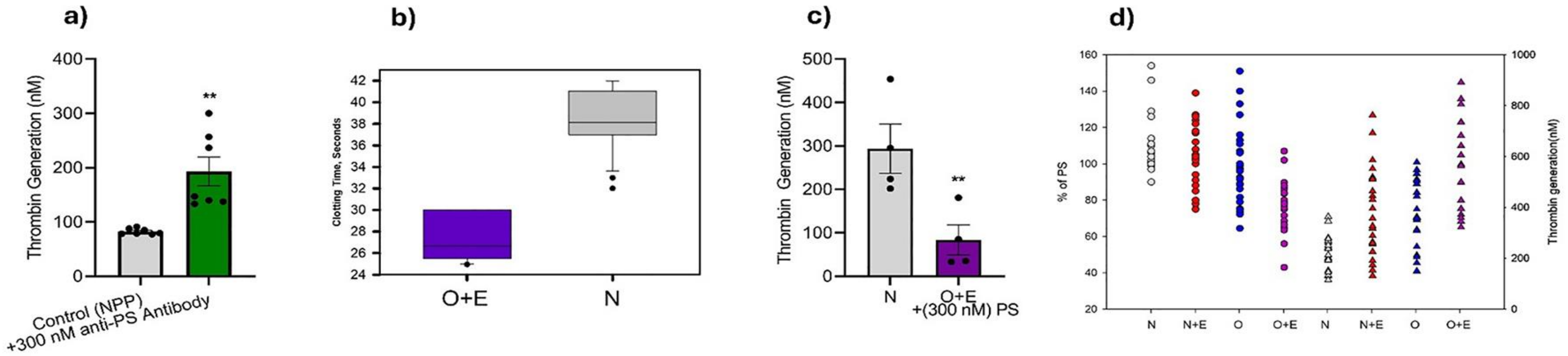

Supplemental Figure 1. The influence of PS on thrombin generation and clotting time in human plasma. TGA was performed to determine the effect of PS on thrombin generation by supplementation with 300 (nM) anti-PS antibody (**a**). Comparison of normal range clotting time between normal (N) vs. obese+estrogen (O+E) plasma by aPTT (**b**). Enhanced thrombin generation in (O+E) plasma by supplementation with 300 (nM) PS (**c**). The ratio of free PS and thrombin formation in human plasma was determined in respect to both estrogen and obesity with a Pearson correlation coefficient value of -0.3782 (**d**). Circle : % of PS. Triangle : Thrombin generation. Kruskal–Wallis test (n=3* p< 0.05. ** p, < 0.01).

Supplemental Figure. 2

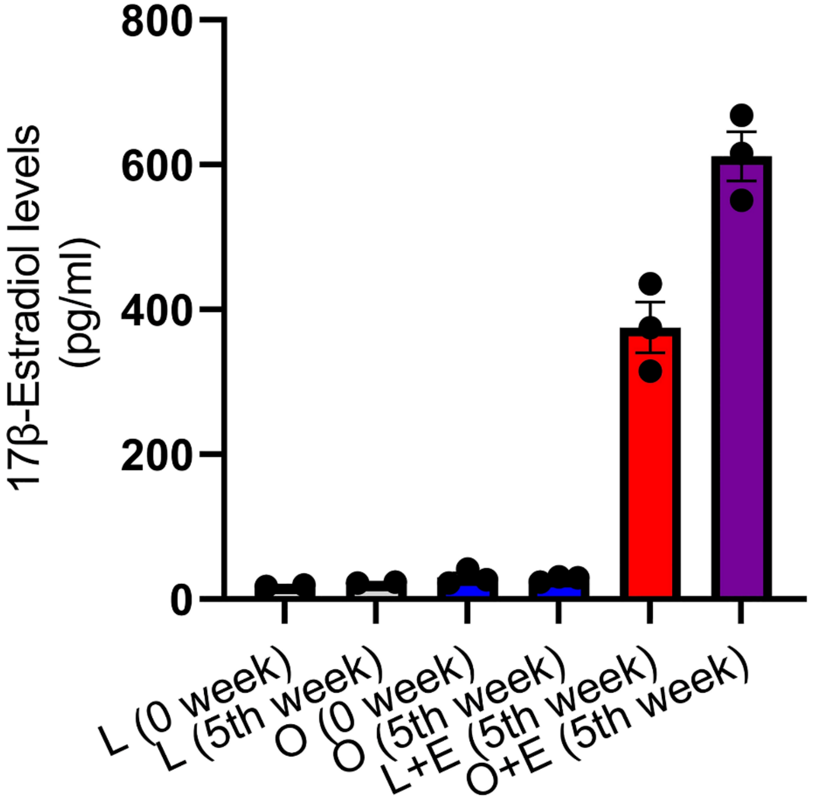

Supplemental Figure 2. Quantitative comparison of 17 β-estradiol levels in obese ± estrogen mice plasma at week 0 and week 5, Kruskal–Wallis test (n=3).

Supplemental Figure. 3

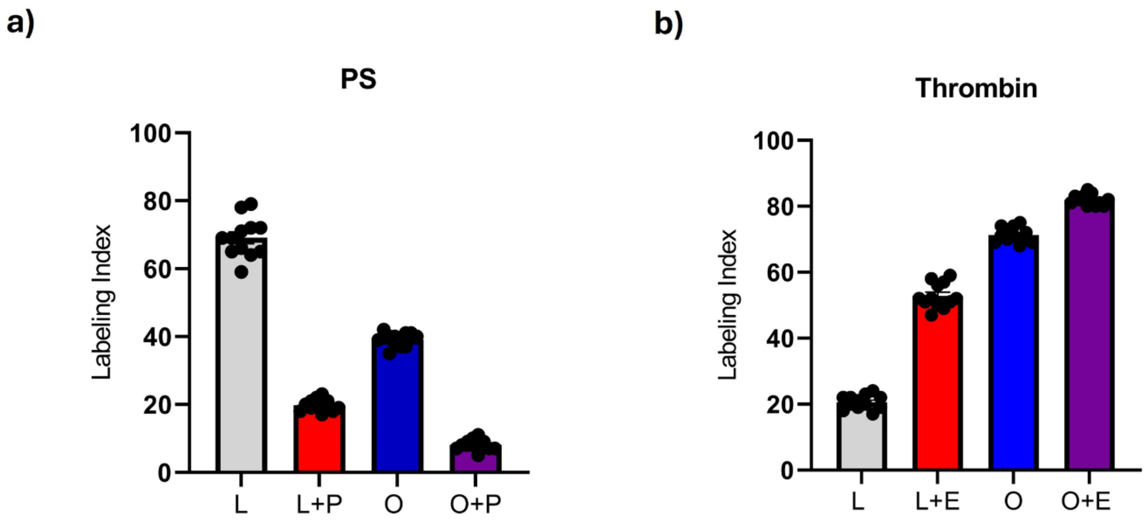

Supplemental Figure 3. Immunohistochemistry quantification of PS and thrombin staining in obese ± estrogen mice liver. Images were processed manually by an expert pathologist.

Supplemental Figure. 4

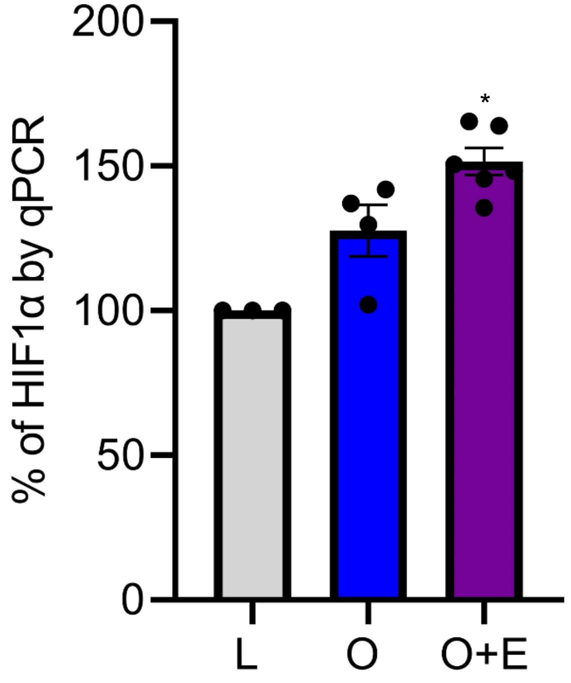

Supplemental Figure 4. The expression level of HIF1α mRNA was quantified by RT-qPCR to determine the effects of obesity and estrogen in obese ± estrogen mice liver, Kruskal–Wallis test (n=3 * $p < 0.05$).

Supplemental Figure. 5

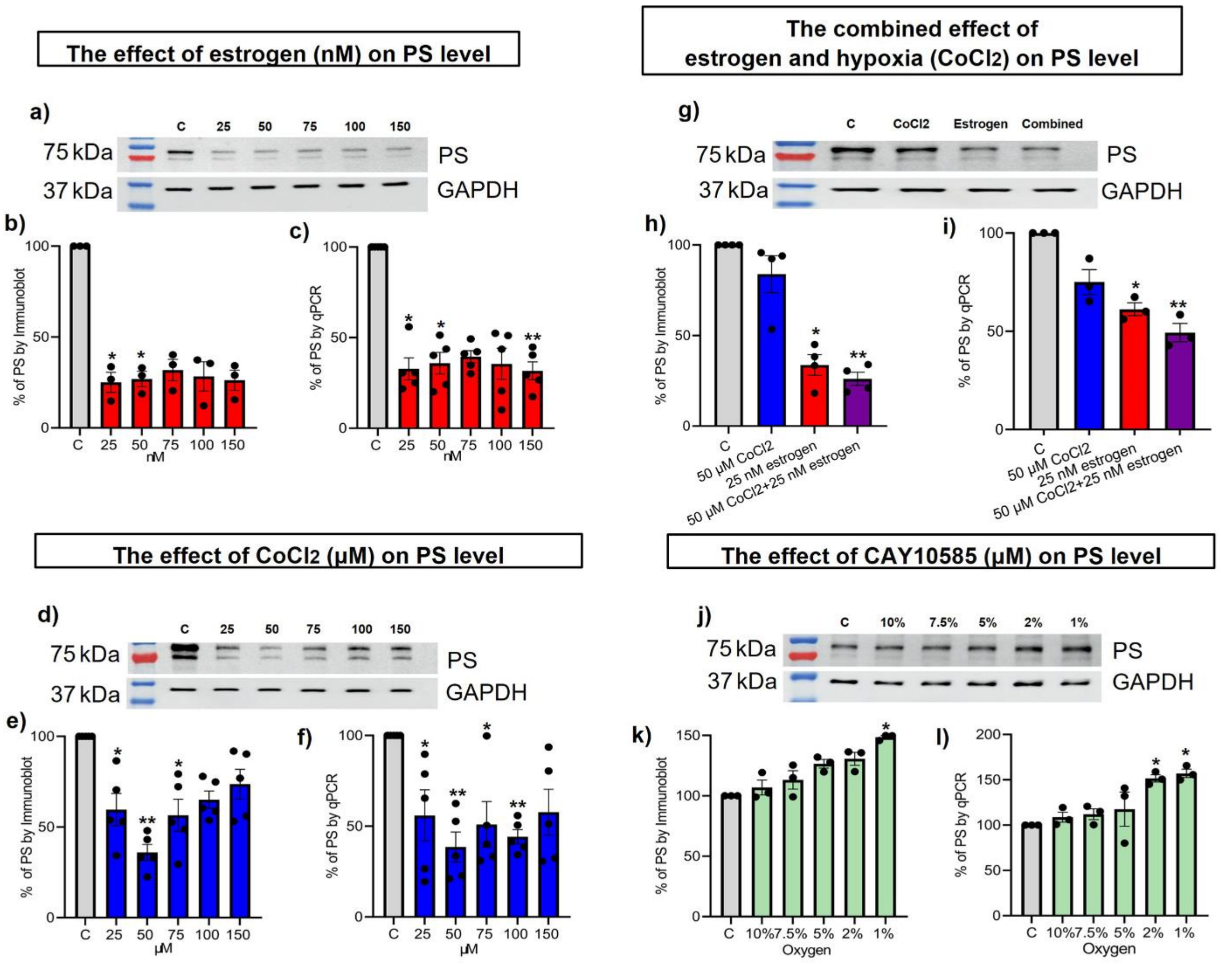

Supplemental Figure 5. The effects of $CoCl_2$ and estrogen on PS downregulation. Estrogen-treated (25-150 nM)/hypoxia-induced (25-150 μM) HepG2 cells were lysed and separated by SDS-PAGE and immunoblot membranes were probed with either PS or GAPDH to determine the effects on PS levels (**a** and **d**). Quantification of relative PS determined by Immunoblot (**b** and **e**) and RT-qPCR (**c** and **f**). The combined effects of (25 nM) estrogen and (50 μM) $CoCl_2$ on PS expression levels were quantified (**g**) and relative PS was determined by immunoblot (**h**) and RT-qPCR (**i**). The reverse effect of 30 (μM) CAY10585 on PS level was quantified (**j**) and relative PS was determined by immunoblots (**k**) RT-qPCR (**l**). The bands were quantified by ImageJ software. The values shown are the mean ± SD of at least three independent experiments. Statistical significance was performed by one-way ANOVA with Kruskal–Wallis correction since data do not follow a normal distribution (* $p< 0.05$. ** p, < 0.01).

Supplemental Figure. 6

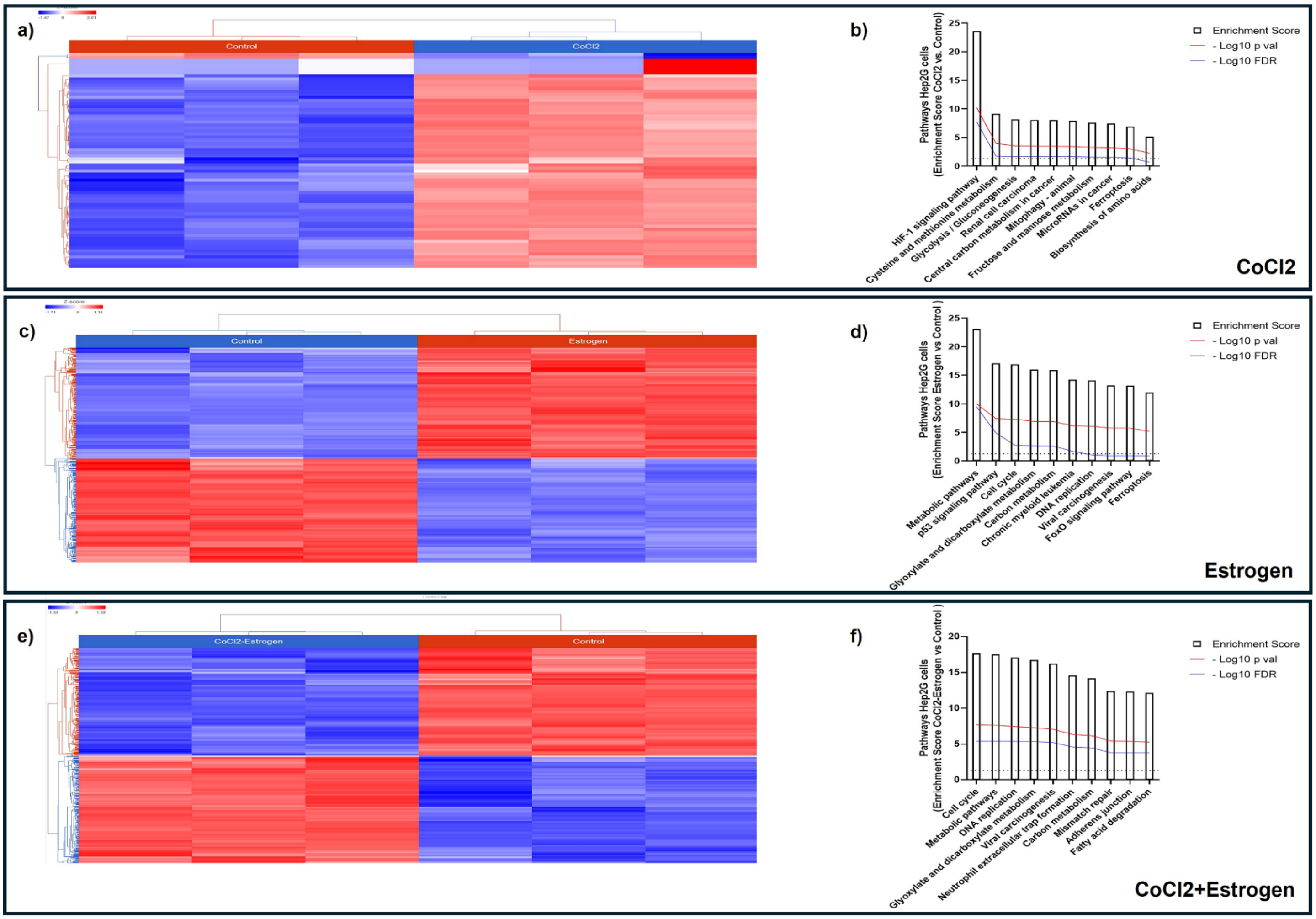

Supplemental Figure 6. Differentially expressed genes related to $CoCl_2$ and estrogen treatment. Heat map of differentially expressed genes in $CoCl_2$ vs. control in HepG2 cells (**a**). Enrichment score of top 10 pathways in $CoCl_2$-treated cells vs. control (**b**). Heat map of differentially expressed genes in estrogen vs. control (**c**). Enrichment score of top 10 pathways in estrogen-treated cells vs. control (**d**). Heat map of differentially expressed genes in $CoCl_2$-estrogen treated cells vs. control in HepG2 cells (**e**). Enrichment score of top 10 pathways in $CoCl_2$-estrogen treated cells vs. control (**f**). Statistical significance of FDR ≤ 0.05.

Supplementary Methods

Thrombin generation assay:

A total of (40 µl-human/20 µl-mice) of plasma was loaded into 96-well microplate followed by (20 µl-human/40 µl-mice) of TGA Buffer (50 mM Tris, 175 mM NaCl, 0.6% PEG-8000, pH 7.4).10 µl of Technothrombin RB reagent (DiaPharma; 5006209, OH, USA) that contained a low concentration (0.4 pmol/L) of tissue factor and 8 µmol/L phospholipid was added, and the solution was incubated at 37°C for 5 min (Supplemental Table 2). The reaction was triggered by the addition of the fluorogenic substrate Z-Gly-Gly-ArgAMC (DiaPharma; 5006230), and fluorescence was measured using a SpectraMax plate reader for 90 min with a time interval of one minute. The emission and excitation wavelengths are 360 and 460 nm, respectively.

Modified activated partial thromboplastin time assay:

A total 40 µl of plasma was loaded into a 96-well microplate followed by 40 µl of Pacific Hemostasis™ KONTACT reagent (Fisher Scientific; 100308, NH, USA). The microplate was incubated for 3 minutes at 37°C, and the reaction was initiated by the addition of 40 µl of (25 mM CaCl2). The absorbance was measured at 405 nm using a plate reader.

Protein expression via immunoblotting

To ensure that we yield the purest protein form, the isolated human plasma was purified using HAS/immunoglobulin Depletion Mini Spin Column (Thermo Fisher Scientific; A36366, MA, USA) while mice plasma was purified using Multi Affinity Removal Spin Cartridge Mouse-3 (Agilent Technology Santa Clara; 5188-5289, CA USA) according to the manufacturer’s instruction. The purified protein was quantified by Bradford assay (Thermo Fisher Scientific; 23238, MA, USA). Human (20 µg), mice (15 µg) and cell (10 µg) samples were lysed in Laemmli buffer (2% w/v SDS, 10% v/v glycerol, 5% v/v 2-mercaptoethanol, 0.002% w/v bromophenol blue and 62.5 mM Tris-

HCl, pH 6.8) and heated for 5 min at 95°C to denature the proteins. The samples were then electrophoresed in SDS-PAGE and the separated proteins were transferred onto a nitrocellulose membrane (Bio-Rad; 1620145, CA, USA). The membrane was washed with Tris-buffered saline with 0.1% Tween, blocked with 5% non-fat dry milk for 1 hour, probed with the following antibodies (Supplemental Table 4). For mice, Protein S antibody (2μg/ml; R&D Systems; MAB4976, MN, USA). For human, sheep anti-human Protein S (0.5 mg; Enzyme Research Laboratories; SAPS-AP, IN, USA); and for cells, anti-Protein S antibody (0.481 mg/ml; Abcam; ab280885, MA, USA). For normalization, GAPDH (GeneTex; GT239, CA, USA) and Eef2 (Abcam; ab33523) antibodies were used. All antibodies were diluted at 1:500 in a blocking solution, and incubated overnight at 4°C. On the following day, the membranes were washed three times with TBST and incubated with alkaline phosphatase (AP)-conjugated antibodies directed against the species raised in the primary antibodies. The band densities were quantitated by ImageJ V1.51 software.

Chromatin immunoprecipitation assay:

HepG2 ($1x10^6$) cells were seeded out into a 6-well plate and allowed to attach overnight. The following day, the cells were washed 2x with PBS and incubated with serum-free media overnight. To assess the combined effects of estrogen and hypoxia, the cells were treated with β-Estradiol (25 nM) and CoCl2 (50 μM) for 4 hours. ChIP was performed using the ChIP-IT Express Enzymatic Kit (Active Motif; USA) according to the manufacturer’s instruction. The cross-linked and sheared chromatin was precipitated with 5 μg of HIF1α and isotype control IgG (Cell Signaling; 14179S and 2729S, MA, USA) antibodies (66, 67). The precipitated DNA was amplified using mice (68) and human primers as listed in (Supplemental Table 3).

Animal model:

All animal procedures were performed according to protocols approved by the Institutional Animal Care and Use Committee of LSU Health New Orleans. 6-week old C57BL/6 female mice were purchased from Charles River laboratory (strain no. 000664) and were blindly randomized into 4 groups with 10 mice in each group as follows: lean (L), lean+estrogen-pelleted (L+P), obese (O), and obese+estrogen-pelleted (O+P). Two groups were fed a normal chow diet while the other two groups were fed a high fat diet (HFHSD; 29% Fat Kcal TD 07011, Inotiv; Teklad, USA) for 3 months, and the body weight of each mouse was recorded until the experiments were completed. The obese mice were administered with a subcutaneous 17 β Estradiol tablet 800 µg/mouse (Innovative Research of America; E-121, FL, USA). The plasma was collected retro-orbitally each week for further assays (69). After the 5th week, the mice were sacrificed, and the livers were also collected for further analysis.

Quantitation of human and mice PS by ELISA:

Human plasma was isolated and quantified using the REAADS Free Protein S Antigen Test Kit (DiaPharma; K051-001, OH, USA) according to the manufacturer's instructions. To quantitate PS in mice plasma, we used in-house developed sandwich ELISA. The Nunc Maxisorp 96-well microplate was coated with 10 µg/ml (150 µl) of polyclonal rabbit purified IgG anti-protein S (Agilent Dako; A0384, CA, USA) antibody (Supplemental. Table 4).and incubated overnight at 4℃. The plate was blocked with blocker casein (Thermo Fisher Scientific; 37532, MA, USA) at room temperature for 2 hours. The total protein concentration in plasma was quantified using Bradford assay and (300ng) of protein was loaded into each well and incubated for 2 hours at room temperature. The wells were washed three times with TBS, 0.02% Tween, then incubated with (150 µl) monoclonal rat anti-mouse Protein S (0.5 µg/ml) (R&D Systems; MAB4976, MN USA),

for 1 hour at room temperature. The wells were washed again with TBST, then incubated for 1 hour at room temperature with (150μl) HRP-labelled Goat anti-Rat IgG (Abcam; ab97057, MA, USA) diluted 1:35000 in TBST. 100μl of 3,3',5,5'-Tetramethylbenzidine (TMB) Liquid substrate system (Sigma-Aldrich; T8665, MO, USA) was added into each well for 5 min at room temperature and the reaction was stopped by adding 100μl 1M HCL. Furthermore, (L, O, and O+E) mice plasma was quantified using the 17 beta Estradiol competitive ELISA (Abcam; ab108667, MA, USA) according to the manufacturer's instructions.

RT-qPCR assessment of gene expression

HepG2 ($5x10^4$) cells were seeded out into 24-well plates in complete EMEM media and allowed to attach overnight. The following day, the cells were washed 2x with PBS and incubated with serum-free media overnight. To assess the effects of estrogen or hypoxia, the cells were treated with various concentrations of β-Estradiol (25-150 nM) or hypoxic conditions (10%-1%) for 4 hours, respectively. To assess the synergistic effects, the cells were treated under hypoxic conditions (10%-1%) with β-Estradiol (25 nM) for 4 hours. To reverse the effect of estrogen, 30 nM Fulvestrant (Sigma-Aldrich; I4409-25MG) was added into cells incubated in serum-free media for 4 hours. Further, to reverse the effect of hypoxia, cells incubated with serum-free media were induced by hypoxia for one hour followed by 30 μM CAY10585 (Abcam; ab144422) for 4 hours. The cells were then washed 1x with ice-cold PBS and immediately lysed according to the manufacturer's instructions (Qiagen; 74104, MD, USA). The RNA concentration was measured via NanoDrop (Thermo Fisher Scientific, Inc.). The cDNA was then synthesized using iScript cDNA Synthesis Kit according to the manufacturer's instructions (Bio-Rad; 1708891, CA, USA), and the amplification reaction was carried out on (Applied Biosystems Veriti™ Thermal Cycler, USA) by using SsoAdvanced Universal SYBR Green Supermix (Bio-Rad; 1725271, CA, USA)

with the following *PROS1*, *HIF1A* or *GAPDH* primers as listed in (Supplemental Table 3). *PROS1* or *HIF1A* gene expression levels were normalized to the expression level of *GAPDH*, and the fold change was calculated using 2−ΔΔCt method.

Effect of HIF1α and ERα on PS promoter activity

To examine the synergistic effects of hypoxia and estrogen on the PS expression, an 800-bp segment of the PS gene promoter (Supplemental Table 3) was cloned into a luciferase reporter vector pGL4 (Promega; E675A, WI, USA) and the clone isolated and prepared according to the manufacturer's instruction (Qiagen; Hilden, Germany). HepG2 ($9x10^4$) cells were seeded out into 12-well plates and allowed to attach overnight. The following day, the cells were washed with PBS and then transfected with 0.5 µg/well of pGL4-PS DNA for 36 hours. The cells were incubated under hypoxic conditions (10%-1%) with β Estradiol (25 nM) for 4 hours, and the promoter activity was analyzed by detecting firefly luciferase activity according to the manufacturer's instructions (Promega; E1500, WI, USA).

Histology & Immunohistochemistry

Isolated mice liver tissues were fixed in 10% buffered formalin and embedded in paraffin. Sections of 4 microns in thickness were cut and placed on electromagnetically charged slides (Fisher Scientific; 22-170-333, NJ, USA) to prevent detachment. Hematoxylin and Eosin were performed for routine histopathological examination. Fibrin was stained with a Martius Scarlet Blue (MSB) special stain kit, following the manufacturer's protocol (ScyTek Laboratories; MSB-2, UT, USA). Immunohistochemistry was performed using Vectastain ABC Elite Kit, according to the manufacturer's instructions (Vector Laboratories; PK-6100, CA, USA). Our modified protocol includes deparaffination in 2 changes of xylenes, rehydration through descending grades of ethanol up to water, non-enzymatic antigen retrieval with 0.01 M sodium citrate buffer pH 6.0 at

95°C for 25 min in a vacuum oven, endogenous peroxidase quenching with 3% H_2O_2 in methanol, blocking with normal goat serum and incubation with the following antibodies (Supplemental Table 4) anti-Protein S (1:200 dilution, Bioss; bs-9512R, MA, USA), and anti-Thrombin (1:100 dilution, Invitrogen; PA5-99213, MA, USA) overnight at room temperature in a humidified chamber. The sections were washed with PBS three times, and incubated with biotinylated secondary antibodies for 1 hour, followed by incubation with avidin-biotin-peroxidase complexes for 1 hour, both at room temperature in a humidified chamber. Finally, the peroxidase was developed with diaminobenzidine (Boehringer, Mannheim, Germany) for 3 minutes, and the sections were counterstained with Hematoxylin and mounted with Permount (Fisher Scientific Fisher Scientific, NH, USA). Photomicrographs were taken with an Olympus DP72 Digital Camera using an Olympus BX70 microscope (Olympus, PA, USA).

RNA library preparation, sequencing and analysis

RNA quantification was performed using the Qubit RNA HS Assay kit (Invitrogen; Q32855, MA, USA) and RNA quality was assessed with the Agilent 2100 bioanalyzer (Agilent Technologies, CA, USA). The libraries were generated using Illumina Stranded Total RNA prep with Ribo-Zero Plus library preparation kit (Illumina; 20040525, CA, USA) according to manufacturer's instructions. Briefly, ribosomal RNA was first depleted from total RNA (100 ng). Following purification, the RNA was fragmented, and first- and second-strand cDNA synthesized. Libraries were created from the cDNA by first adenylating the 3′ end and ligating anchors to the ends. An amplification step of 13 cycles of PCR was then performed to add unique indexes to the ligated products. Resulting libraries were quantified using the Qubit dsDNA High Sensitivity Assay Kit (Invetrogin; Q32851, MA, USA) and the size and purity assessed with the Agilent 2100 bioanalyzer. The sequencing was performed on Illumina's NextSeq 2000 instrument using a NextSeq 2000 P3 200 cycles reagent kit with pair-end 76 bp reads (Illumina, CA, USA).

FASTQ files were uploaded to Partek Flow for analysis. Contaminants (rDNA, tRNA and mtrDNA) were removed with Bowtie v2.2.5 and the remaining reads were aligned to mm10 using STAR v2.7.8a and quantified with RefSeq Transcripts 96 (release 11/02/2020). The samples were split by attributes (lean vs. estrogen or obese) and analyzed separately. In both instances, features (genes) with 5 or less reads were filtered out and the counts normalized by TMM (+0.0001 / TMM / log2). Differentially expressed genes were identified by using DESEQ2, comparing the counts in the treated samples vs. the control samples. FDR at ≤ 0.05 was applied to all comparisons. Heatmaps, pathways analysis and GO terms were all done in Partek Flow. The sequencing data has been assigned the accession number GSE285380 by GEO.

Supplementary Materials

Supplemental Table 1. List of OCP used by female participants

Sample No	Oral contraceptive Pill (OCP) for N+E participants	Oral contraceptive Pill (OCP) for O+E participants
**1**	Blisovi24FE norethindrone (progestin)+Ethinyl estradiol (estrogen)	Estrogen (ethinyl estradiol 20 µg)+progestin (drospirenone 3 mg)
**2**	Norethindrone (progestin)+Ethinyl estradiol (estrogen)	Estrogen
**3**	Estrogen	Estrogen
**4**	Norethindrone (progestin)+Ethinyl estradiol (estrogen)	Estrogen
**5**	Norgestimate (progestin, synthetic progestogen)+Ethinyl estradiol (estrogen)	Estrogen
**6**	Norgestimate (progestin, synthetic progestogen)+Ethinyl estradiol (estrogen)	Estrogen
**7**	Levonorgestrel (progestin - synthetic progesterone)+Ethinyl Estradiol (estrogen)	Estrogen
**8**	Norethindrone acetate (progestin - synthetic progesterone)+Ethinyl estradiol (estrogen)	Estrogen - Ethinyl estradiol
**9**	Norgestimate (progestin - synthetic progestin)+Ethinyl estradiol (estrogen)	Progestin+Estrogen
**10**	Norethindrone (progestin - synthetic progesterone)+Ethinyl estradiol (estrogen)	Norethindrone (progestin)+Ethinyl estradiol (estrogen)
**11**	Norgestimate (progestin - synthetic progestin) + Ethinyl Estradiol (estrogen)	Norethindrone (progestin)+Ethinyl estradiol (estrogen)
**12**	Drospirenone (progestin- synthetic progesterone)+Ethinyl estradiol (estrogen)	Norethindrone (progestin)+Ethinyl estradiol (estrogen)
**13**	Norethindrone (progestin - synthetic progesterone)+Ethinyl estradiol (estrogen)	Norgestimate (progestin, synthetic progestogen)+Ethinyl estradiol (estrogen)
**14**	Ethinyl estradiol+Levonogestrel	Norethindrone acetate (progestin - synthetic progesterone)+Ethinyl estradiol (estrogen)
**15**	Drospirenone (progestin- synthetic progesterone)+Ethinyl estradiol (estrogen)	Norethindrone (progestin - synthetic progesterone)+Ethinyl estradiol (estrogen)
**16**	Norethindrone (progestin - synthetic progesterone)+Ethinyl estradiol (estrogen)	Norgestrel+Ethinyl Estradiol
**17**	Norethindrone (progestin - synthetic progesterone)+Ethinyl estradiol (estrogen)	Norethindrone (progestin - synthetic progesterone)+Ethinyl estradiol (estrogen)
**18**	Norgestimate (progestin - synthetic progestin)+Ethinyl estradiol (estrogen)	Levonorgestrel (progestin - synthetic progesterone)+Ethinyl Estradiol (estrogen)
**19**	Norethindrone (progestin - synthetic progesterone)+Ethinyl estradiol (estrogen)	Norgestimate+Ethinyl Estradiol (ESTARYLLA) 0.25 mg/0.035 mg; Estrogen
**20**	Drospirenone (progestin- synthetic progesterone)+Ethinyl estradiol (estrogen)	Estrogen
**21**	Drospirenone-Ethinyl estradiol (YAZ) 3-0.02mg; Estrogen	Drospirenone (progestin- synthetic progesterone)+Ethinyl estradiol (estrogen)
**22**	Norgestimate-Ethinyl Estradiol (Ortho TRI-cyclen LO) 10.18/10215/0.25mg-25mcg tablet	Etonogestrel (synthetic progesterone)+Ethinyl estradiol (synthetic estrogen)
**23**	Norethindrone-ethinyl estradiol (MICROGESTIN 1/20)	Estrogen
**24**	Estrogen	Norethindrone (progestin)
**25**	Norgestimate (progestin - synthetic progestin)+Ethinyl Estradiol (estrogen))+Levonorgestrel (progestin- synthetic progesterone	Norethindrone (progestin)
**26**	Estrogen	Estrogen
**27**	Norethindrone (progestin)+Ethinyl estradiol (estrogen)	Estrogen
**28**	Norgestimate (progestin, synthetic progestogen)+Ethinyl estradiol (estrogen)	Estrogen
**29**		Ethinyl estradiol+Progestin (Norethindrone acetate)
**30**		Estrogen
**31**		Estrogen
**32**		Progestin
**33**		Norethindrone (progestin)
**34**		Norethindrone (progestin)
**35**		Progestin
**36**		Estrogen
**37**		Estrogen
**38**		Estrogen
**39**		Estrogen

Supplemental Table 2. kits and reagents

Kits	Manufacturer (Cat. No.)
DNeasy Blood and Tissue Kits for DNA Isolation	Qiagen (69504)
RNeasy Mini Kit	Qiagen (74104)
iScript cDNA Synthesis Kit	Bio-Rad (1708891)
ChIP-IT Express Enzymatic Kit	Active Motif (53009)
Luciferase Assay kit	Promega (E1500)
REAADS® Monoclonal Free Protein S ELISA kit	Diapharma (K051-001)
Martius Scarlet Blue (MSB) Stain Kit	ScyTek Laboratories (MSB-2)
Vectastain ABC Elite Kit	Vector Laboratories (PK-6100)
Qubit RNA HS Assay kit	Invitrogen (Q32855)
Illumina Stranded Total RNA prep, ligation with Ribo-Zero Plus library preparation kit	IIlumina (20040525)
Qubit dsDNA High Sensitivity Assay Kit	Invetrogin (Q32851)
17 beta Estradiol ELISA kit	Abcam (ab108667)
Technothrombin RB reagent	DiaPharma (5006209)
fluorogenic substrate Z-Gly-Gly-ArgAMC	DiaPharma (5006230)
Pacific Hemostasis™ KONTACT reagent	Fisher Scientific (100308)

Supplemental Table 3. RT-qPCR and PCR primers

Method	Gene	Forward primer	Reverse primer	Manufacturer
Luciferase assay	*PROS1*	GGTACCGCTCAGAAGTTCGAGGCTG	CTCGAGGCTCCCTAGTTGCTGCTTTT	
Chip assay	*PROS1* (human)	CCTAGTGAGACCCTGTCTCGG	CTCCTGTCTCAGCCTCCTG	
	PROS1 (mice)	TTCCGTGTTGGCTCATTCC	TTGGTCTGAGATGGCTTTGACA	(Integrated DNA Technologies)
	Exon (13-14)	GCACAGTGCCCTTTGCCT	CAAATACCACAATATCCTGAGACGTT	
	GAPDH (mice)	TCCCACTCTTCCACCTTCGA	AGTTGGGATAGGGCCTCTCTT	
RT-qPCR	*PROS1*	GGCTCCTACTATCCTGGTTCTG	CAAGGCAAGCATAACACCAGTGC	(Origene)
	GAPDH	GTCTCCTCTGACTTCAACAGCG	ACCACCCTGTTGCTGTAGCCAA	
	HIF1α (mice)	CCTGCACTGAATCAAGAGGTTGC	CCATCAGAAGGACTTGCTGGCT	
	GAPDH (mice)	CATCACTGCCACCCAGAAGACTG	ATGCCAGTGAGCTTCCCGTTCAG	

Supplemental Table 4. Primary antibodies

Method	Antibody	Manufacturer	Dilution
Immunoblotting	PS (mice)	R&D Systems (MAB4976)	1:500
	PS (Human)	Enzyme Research Laboratories (SAPS-AP)	1:500
	PS (cells)	Abcam (ab280885)	1:500
	GAPDH	GeneTex (GT239)	1:500
	Eef2	Abcam (ab33523)	1:500
ELISA (mice)	PS	Agilent Dako (A0384)/ R&D Systems (MAB4976)	10 μg/ml/ 0.5 μg/ml
ChIP	HIF1<br>IgG	Cell Signaling (14179S)<br>(2729S)	7 μg/ml,
IHC	PS<br>Thrombin	Bioss (bs-9512R)<br>Invitrogen (PA5-99213)	1:200<br>1:100
