# Supplementary material for: Estrogen and obesity synergistically suppress protein S via HIF1α, enhancing thrombosis potential
Source: J Clin Invest. 2025 Nov 17;135(22):e193976. doi: 10.1172/JCI193976 (PMC12618064; doi:10.1172/JCI193976)
Supplement: Unedited blot and gel images [file jci-135-193976-s201.pdf]

Full unedited gel for Figure 1

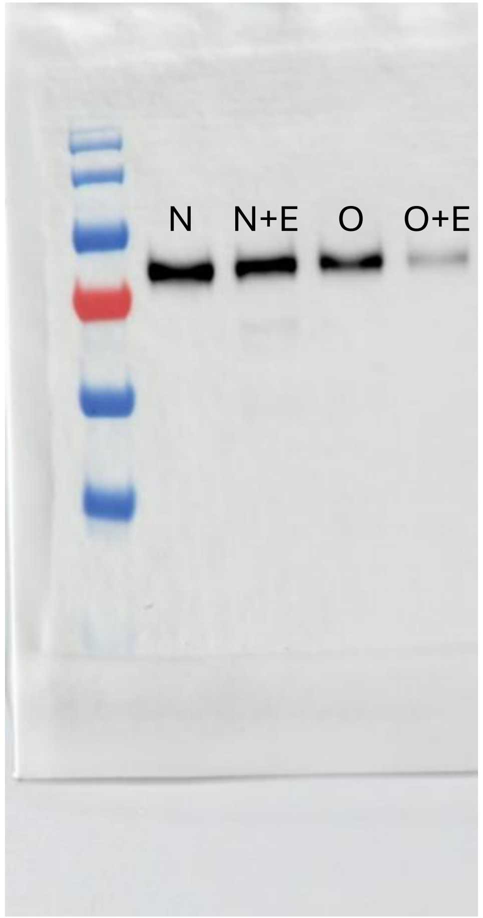

5th week Human plasma samples probed with PS Ab

Full unedited gel for Figure 3

Mice Liver samples probed with PS Ab

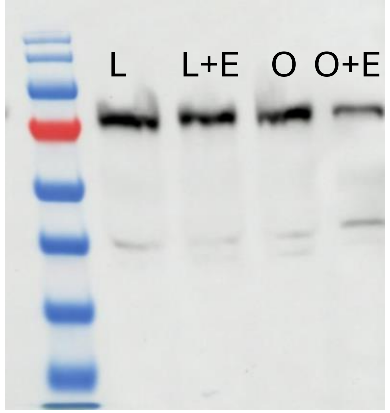

Mice Liver samples probed with Eif 2

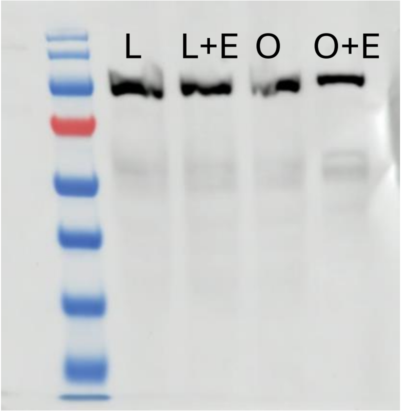

Full unedited gel for Figure 5a

HePG2 cell samples probed with PS Ab

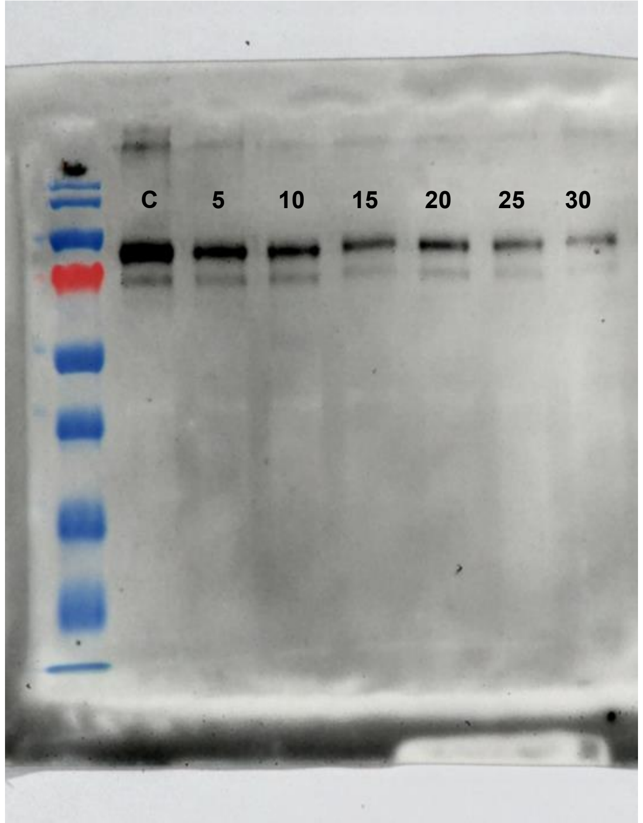

HePG2 cell samples probed with GAPDH Ab

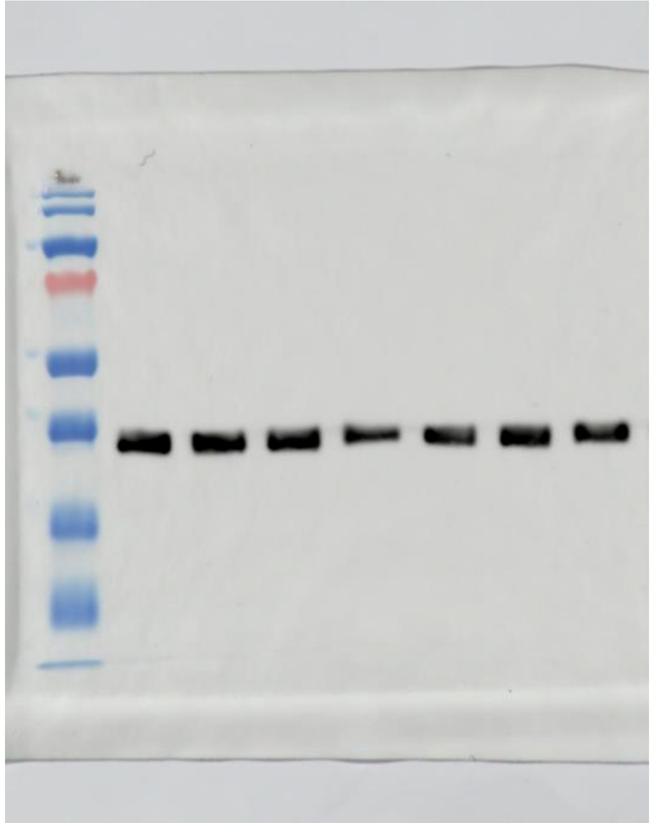

The effect of estrogen 5-30 (nM) on PS expression level

Full unedited gel for Figure 5d

HePG2 cell samples probed with PS Ab

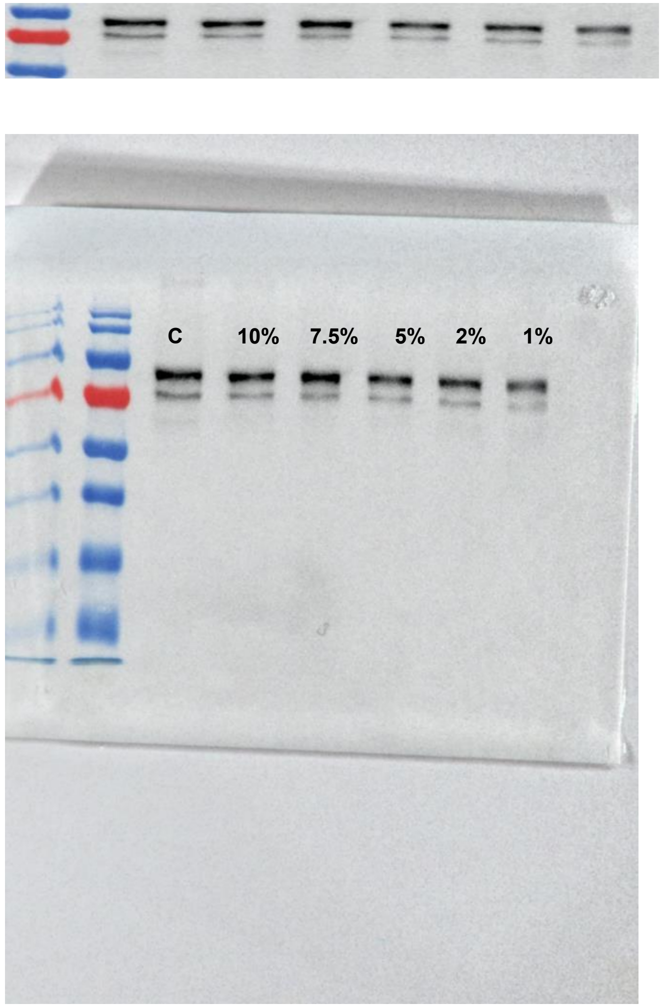

HePG2 cell samples probed with GAPDH Ab

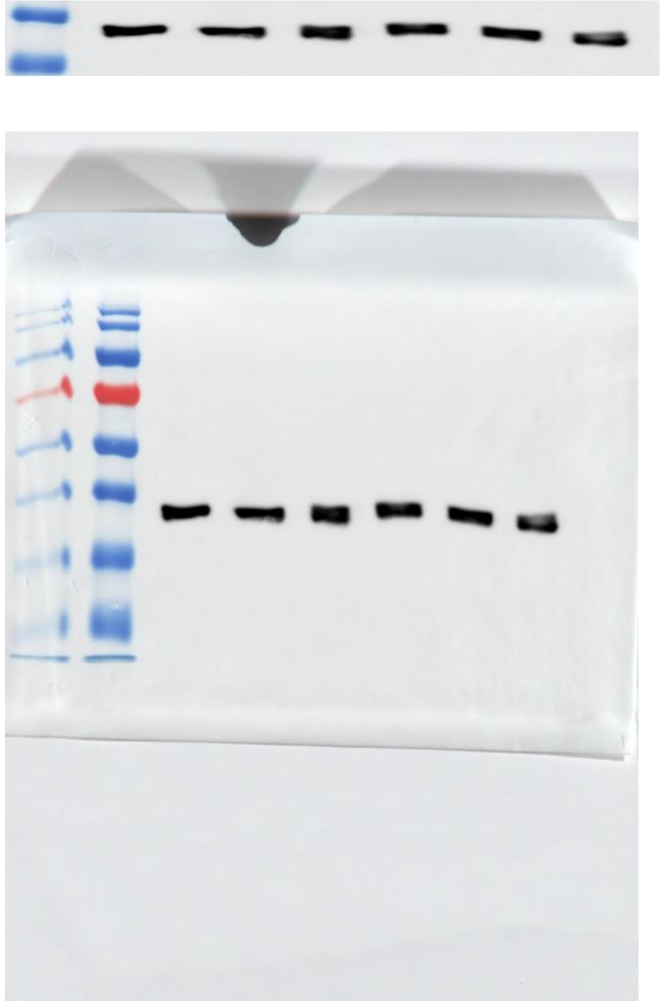

The effect of hypoxia (10%-1%) on PS expression level

Full unedited gel for Figure 5g

HePG2 cell samples probed with PS Ab

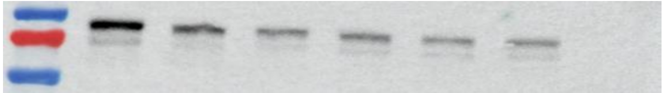

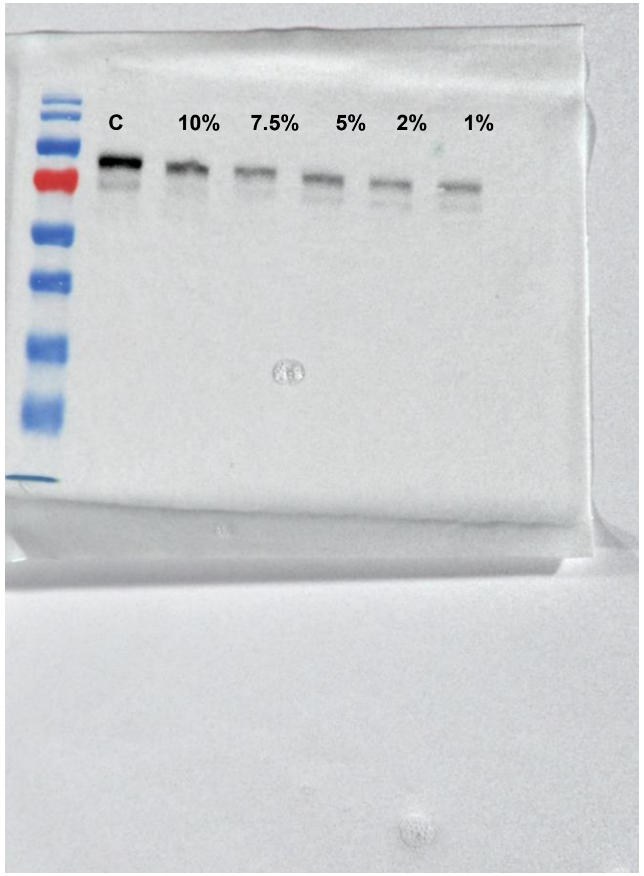

HePG2 cell samples probed with GAPDH Ab

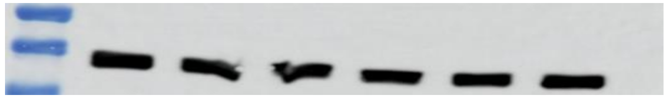

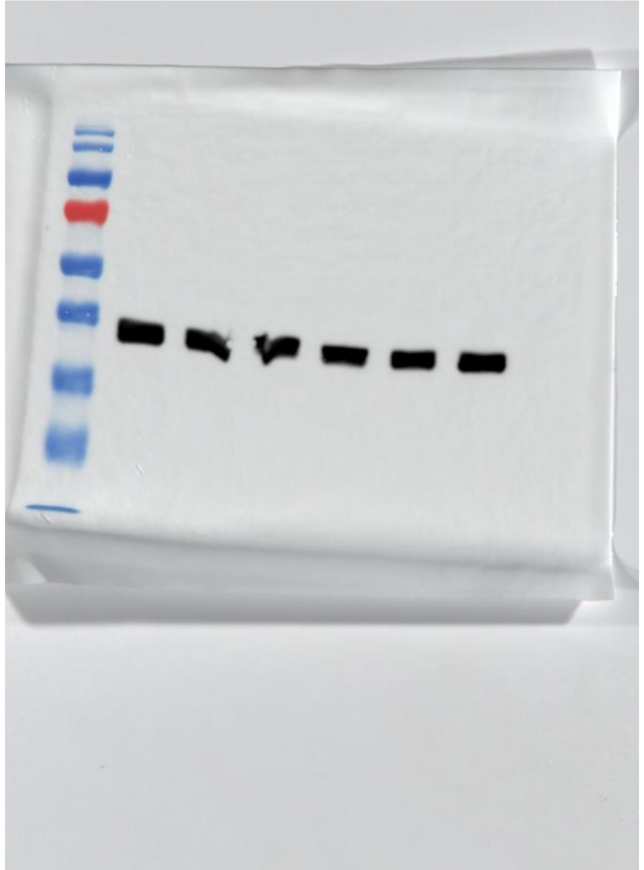

The combined effects of hypoxia (10%-1%) and 25 nM estrogen on PS expression level

Full unedited gel for Figure 5k

HePG2 cell samples probed with PS Ab

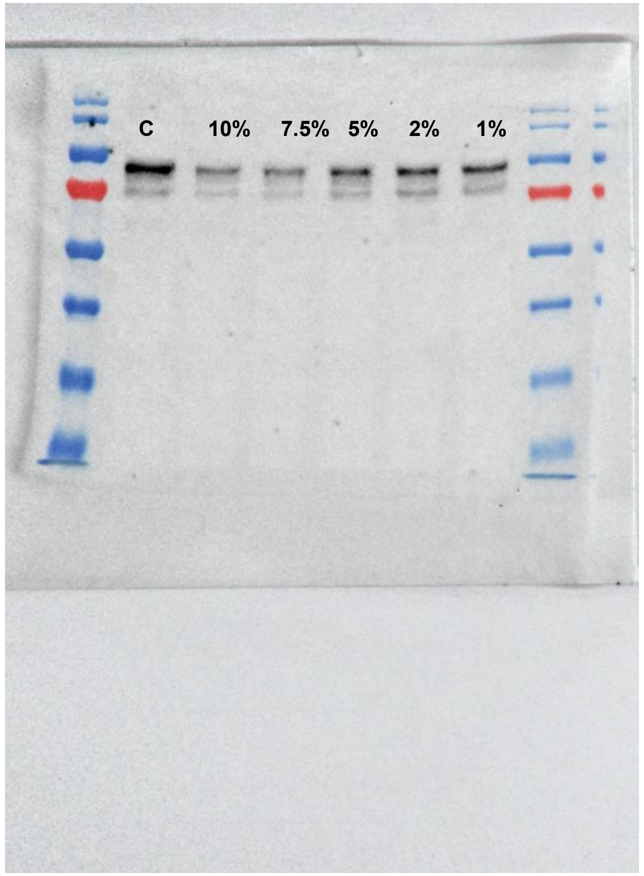

HePG2 cell samples probed with GAPDH Ab

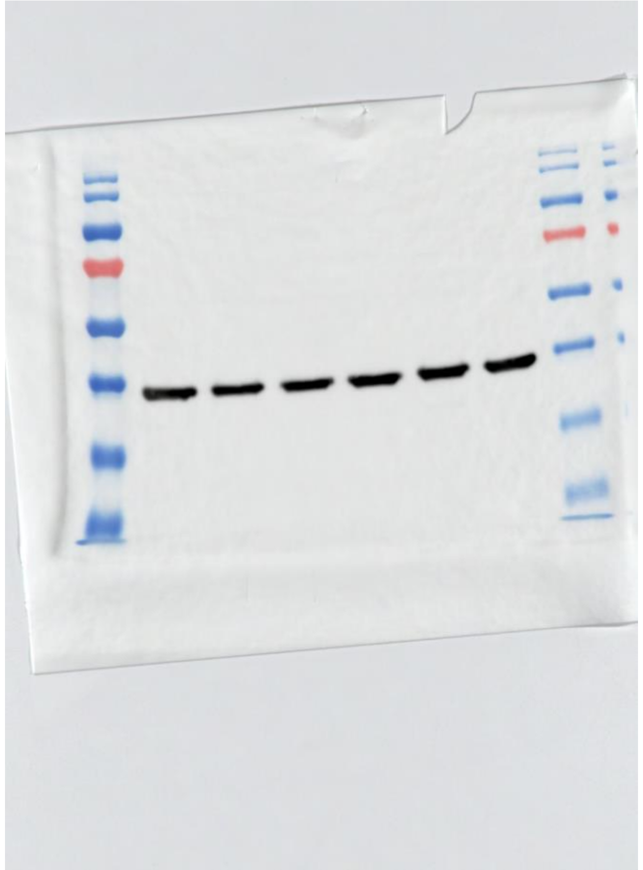

The effect of Fulvestrant 30 (nM) on PS expression level

Full unedited gel for Figure 5p

Chip assay

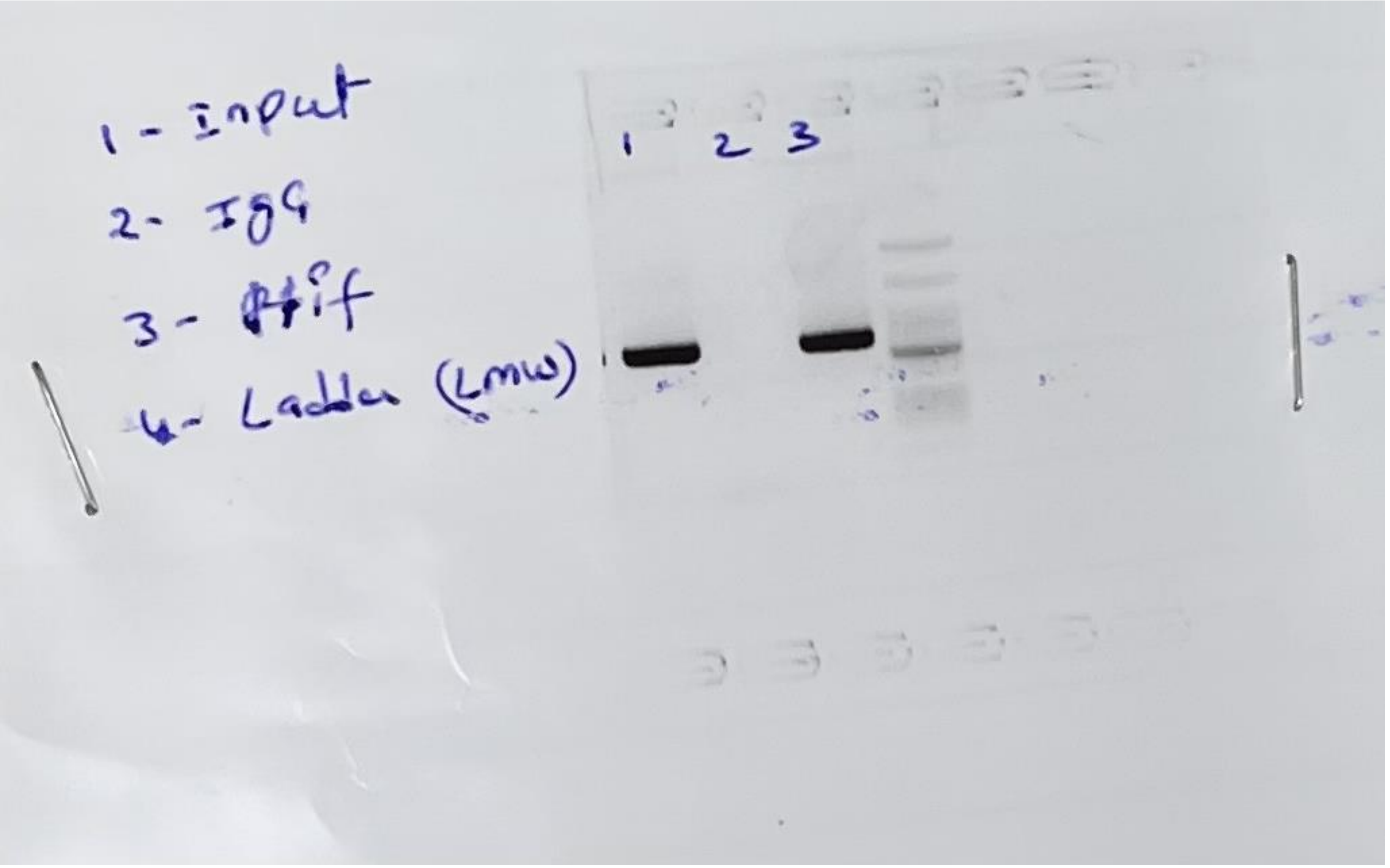

Full unedited gel for Suppl. Figure 6a

HePG2 cell samples probed with PS Ab

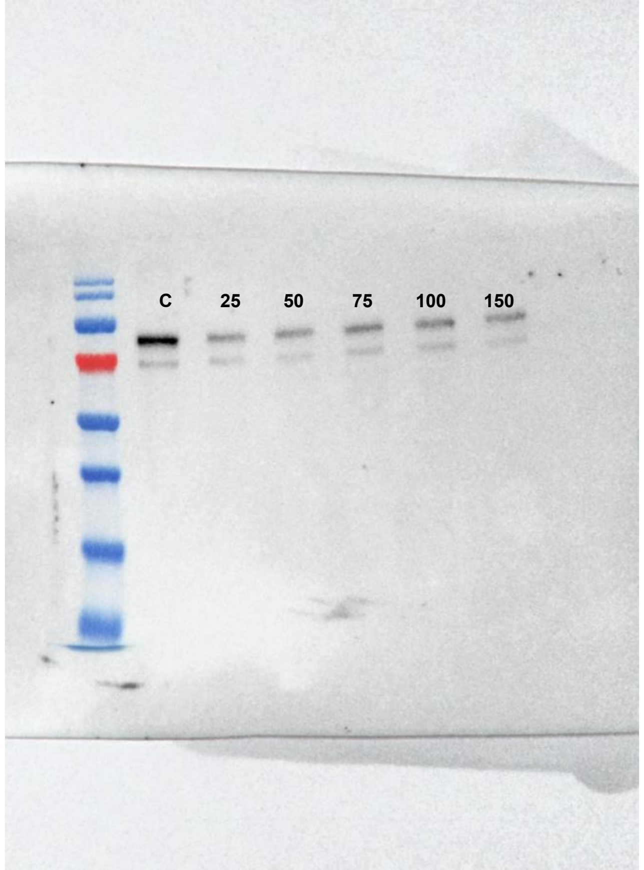

HePG2 cell samples probed with GAPDH Ab

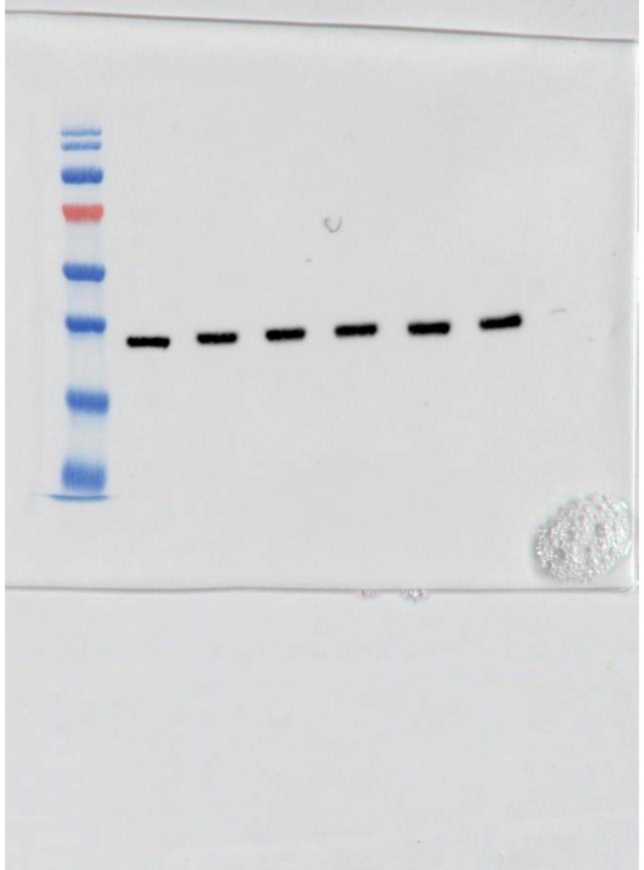

The effect of estrogen 25-150 (nM) on PS expression level

Full unedited gel for Suppl. Figure 6d

HePG2 cell samples probed with PS Ab

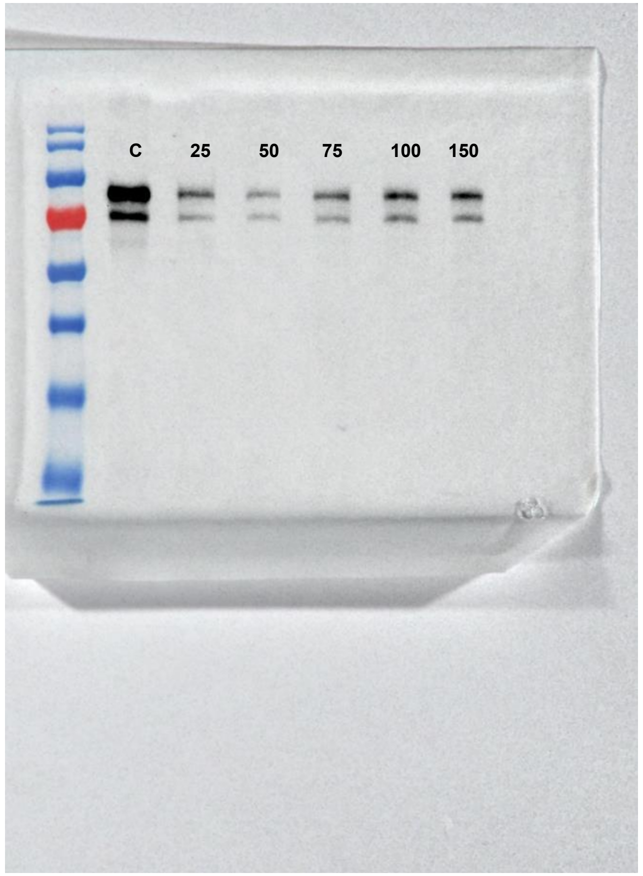

HePG2 cell samples probed with GAPDH Ab

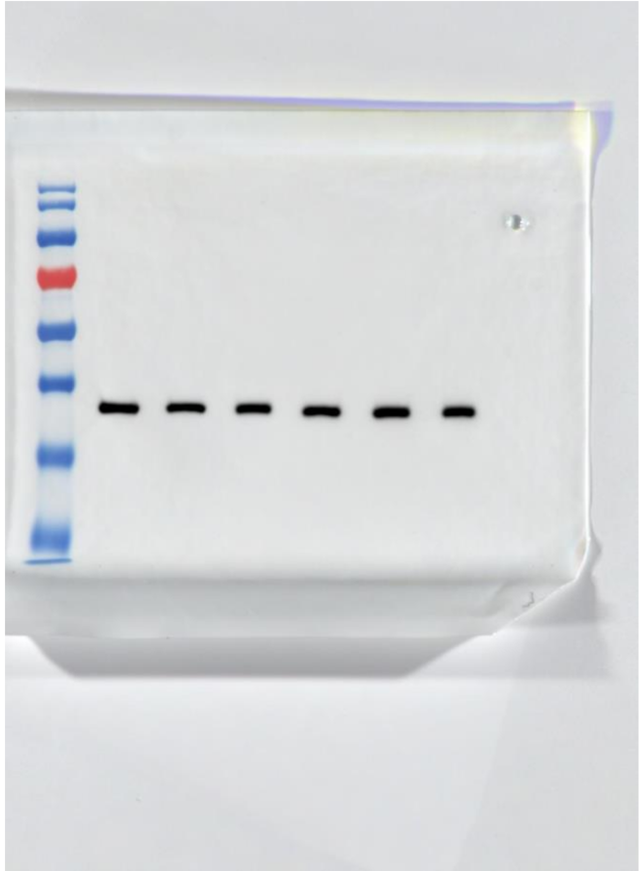

The effects of CoCl2 25-150 (μM) on PS expression level

Full unedited gel for Suppl. Figure 6g

HePG2 cell samples probed with PS Ab

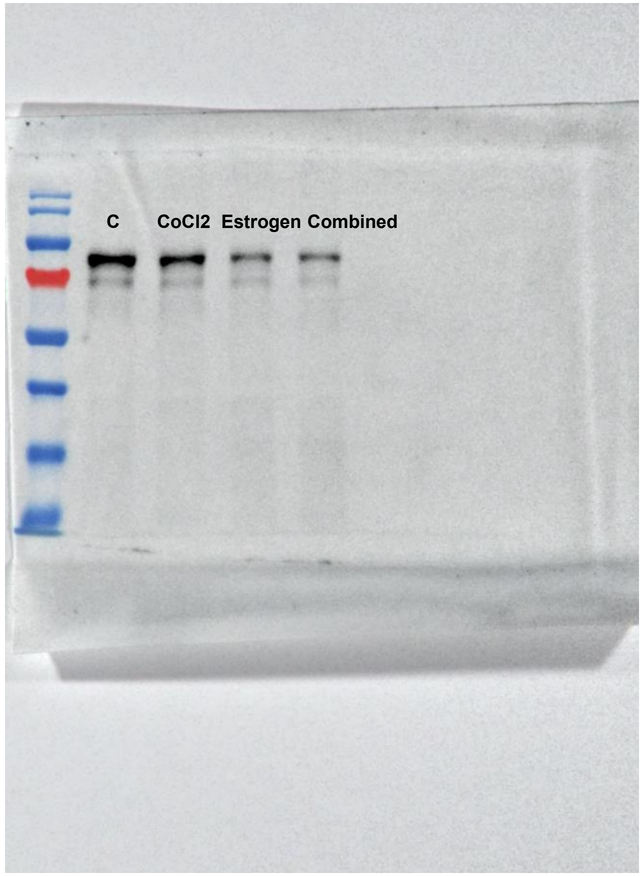

HePG2 cell samples probed with GAPDH Ab

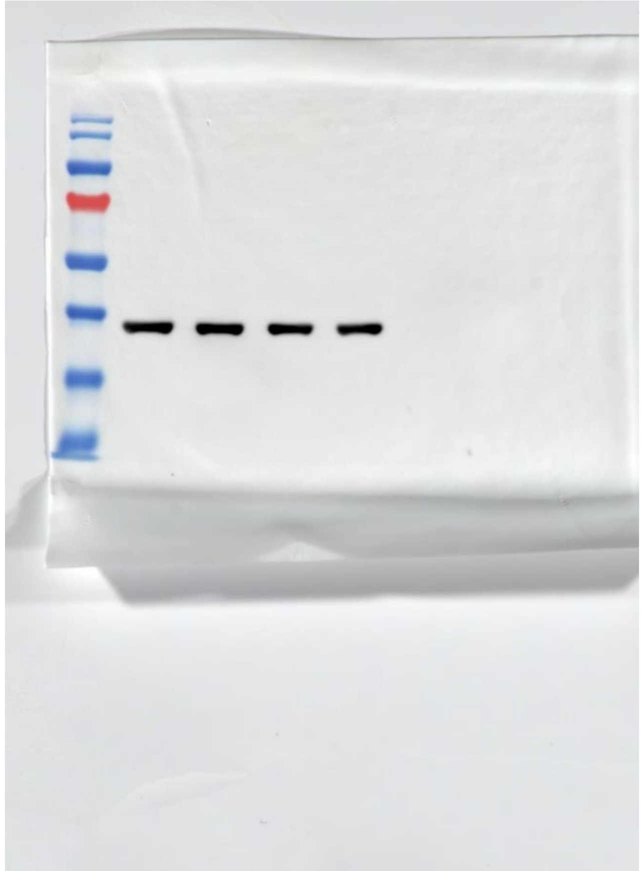

The combined effects of 50 (μM) and 25 (nM) estrogen on PS expression level

Full unedited gel for Suppl. Figure 6j

HePG2 cell samples probed with PS Ab

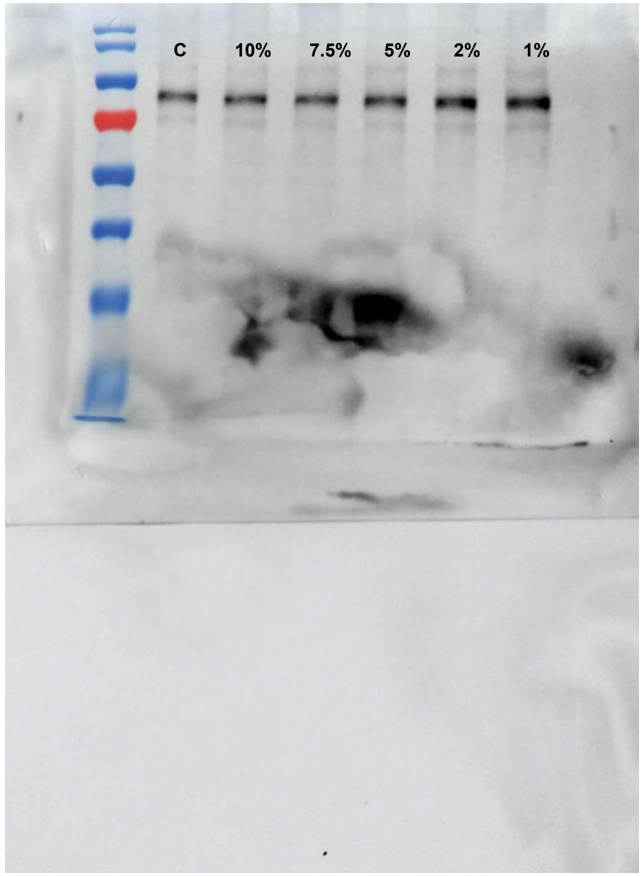

HePG2 cell samples probed with GAPDH Ab

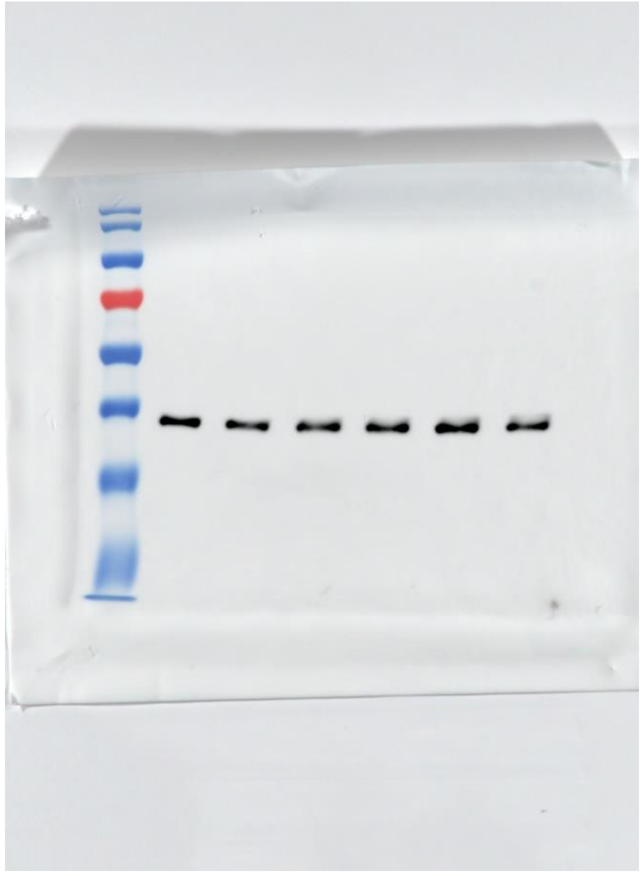

The effects of 30 (μM) CAY10585 on PS expression level
